# Application of MS-Based Metabolomic Approaches in Analysis of Starfish and Sea Cucumber Bioactive Compounds

**DOI:** 10.3390/md20050320

**Published:** 2022-05-12

**Authors:** Roman S. Popov, Natalia V. Ivanchina, Pavel S. Dmitrenok

**Affiliations:** G.B. Elyakov Pacific Institute of Bioorganic Chemistry, Far Eastern Branch of Russian Academy of Sciences, 159 Prospect 100-let Vladivostoku, Vladivostok 690022, Russia; ivanchina@piboc.dvo.ru

**Keywords:** starfish, sea cucumber, polyhydroxysteroids, triterpene glycosides, steroid glycosides, lipids, mass spectrometry, metabolomics, metabolomic profiling

## Abstract

Today, marine natural products are considered one of the main sources of compounds for drug development. Starfish and sea cucumbers are potential sources of natural products of pharmaceutical interest. Among their metabolites, polar steroids, triterpene glycosides, and polar lipids have attracted a great deal of attention; however, studying these compounds by conventional methods is challenging. The application of modern MS-based approaches can help to obtain valuable information about such compounds. This review provides an up-to-date overview of MS-based applications for starfish and sea cucumber bioactive compounds analysis. While describing most characteristic features of MS-based approaches in the context of starfish and sea cucumber metabolites, including sample preparation and MS analysis steps, the present paper mainly focuses on the application of MS-based metabolic profiling of polar steroid compounds, triterpene glycosides, and lipids. The application of MS in metabolomics studies is also outlined.

## 1. Introduction

The emergence of novel diseases and resistant forms of known diseases in recent years and the emergence of multidrug-resistant pathogens has led to a renewed interest in the exploration of new sources of bioactive compounds. The sea environment possesses extraordinary ecological variety, and its inhabitants exhibit enormous biochemical diversity. At present, over 29,000 marine natural products have been discovered [1,2,3,4]. To date, several dozen marine natural products or their derivatives have been approved as therapeutic agents or are undergoing Phase III, II, or I drug development [5]. As the biodiversity of marine organisms is higher than that of terrestrial plants and animals, and as only a minor portion of metabolites present in marine species has been studied, it can be assumed that the number of new marine compounds will continue to increase, providing new therapeutic alternatives.

Marine invertebrates have long been considered an inexhaustible source of novel natural products. Although Porifera and Cnidaria are the two major sources of new marine natural products, Echinodermata is viewed as another abundant source of new bioactive compounds. Over the past five years, just over two hundred new compounds have been isolated from echinoderms [1,2,3,4,6]. The phylum Echinodermata includes about 7500 species found in all seas at every depth, from intertidal to abyssal, and in all ecosystems, from coral reefs to shallow shores. Echinoderms are divided into five different taxonomic classes, including Asteroidea (starfish) and Holothuroidea (sea cucumbers).

Starfish and sea cucumbers are extensively employed in traditional medicine, being a rich source of bioactive compounds. Several starfish species are used to treat rheumatism or as tonics in traditional Chinese medicine [7,8]. Sea cucumbers are one of the most valuable aquaculture species in China, Korea, and Japan, as well as other countries, where they are used as functional foods. Traditional medicine in China and other countries in Asia and the Middle East uses sea cucumbers widely to treat a broad range of diseases, including asthma, arthritis, hypertension, and kidney disease [9,10].

Unique pharmacological properties, including anticancer, antioxidant, antithrombotic, and immunostimulating activities, among others, are associated with bioactive starfish and sea cucumber compounds [11,12,13,14,15]. Moreover, the secondary metabolites of sea cucumbers affect the biological clock and circadian rhythm of lipid metabolism [16], reduce fat accumulation [17], and protect against high fat diet-induced metabolic disorders in mice [18]. Extracts of certain specimens may accelerate wound healing and tissue regeneration [19].

The distinctive chemical composition of starfish and sea cucumbers seems to be the main reason for these beneficial properties. Starfish and sea cucumbers are high in valuable nutrients such as vitamins, minerals, and metabolites such as peptides, sterols, phenolics, sphingolipids, glycosaminoglycans, sulfated polysaccharides, and lectins [10]. Among these compounds the most exciting are unique polar steroid compounds and triterpene glycosides, which are characteristic of starfish and sea cucumbers. These compounds have unusual chemical structures and demonstrate a variety of biological effects, such as cytotoxic, antifungal, antiviral, antibacterial, anti-inflammatory, analgesic, ichthyotoxic, hemolytic, anti-biofouling, anticancer, immunomodulating, and neuritogenic actions [12,20,21,22,23,24,25,26,27,28,29,30,31,32,33].

Secondary metabolites are usually present in the extracts of starfish and sea cucumbers as complex mixtures of very similar compounds. Conventional methods for the structural study of bioactive compounds are usually time-consuming and labor-intensive procedures that include the isolation of individual compounds by a combination of chromatographic techniques and structure elucidation through a combination of different methods [20]. The final structure confirmation of a new compound is always performed with a set of independent methods, such as nuclear magnetic resonance (NMR) spectroscopy, mass spectrometry (MS), or other analytical methods and chemical transformation. Despite the instrumentation developments of recent years, the analysis of bioactive natural compounds using conventional approaches remains a challenging task due to the difficulty of isolating individual compounds from fractions consisting of many components and with high chemical diversity covering a broad concentration range. As a result, only certain compounds can be described; overall, the entire metabolite pool remains poorly studied.

At present, modern mass spectrometry techniques are widely employed for the identification and structural analysis of novel natural compounds [34,35,36]. Hyphenated techniques combining various separation methods with mass spectrometry are applied for metabolomic and target profiling and allow for the characterization of compounds in complex mixtures extracted from biological material. Different MS-imaging techniques precisely localize and quantify the metabolites in tissues [37]. Recently developed ion mobility (IM) methods add dimension to conventional chromatography separation, allowing the stereoisomers that cannot be separated by liquid chromatography (LC) to be identified [38].

Mass spectrometry is used in various fields of marine sciences today, including marine proteomics [39], metabolomics [40,41], lipidomics [42], marine toxicology [43], ecology studies [44], and others. The introduction of modern mass-spectrometric approaches has greatly contributed to the development of metabolomics as a transdisciplinary science that aims at the qualitative and quantitative determination of the whole metabolite pool of organisms. Although no single analytical method exists that can determine all members of the metabolome simultaneously, MS-based metabolomics has been successfully applied to analyze a wide range of compounds from various sources. In recent years, MS-based metabolomics has emerged as a useful tool in natural product research. In addition to metabolic fingerprinting, two approaches used in metabolomics studies can be distinguished [45]. The first, metabolic profiling, focuses on the analysis of structure-related metabolites or metabolites related to a specific metabolic pathway. Such an approach provides information on the chemical composition of extracts or fractions and allows for the dereplication of known bioactive compounds and detection of new compounds as well as the evaluation of the their isolation possibility [34]. The metabolome-oriented approach aims to detect differences in metabolic profiles that occur in response to stress, disease, changing environmental conditions, or other influences in comparative experiments.

Metabolomic studies of marine organisms is a field that uses a variety of modern approaches, including MS, NMR methods, and hyphenated techniques [40,41,44]. This review focuses on the use of MS-based metabolomics techniques applied in studies of extracts and fractions of starfish and sea cucumber bioactive compounds. The following section provides a general overview of the characteristic features in workflows, including sample preparation and analytical approaches. Section 3 includes illustrative examples of MS-based applications in the analysis of bioactive compounds such as polar steroids, triterpene glycosides, cerebroside, and ganglioside, as well as examples of the multi-class approach application. Instrumental and methodological details are highlighted and summarized in tables (Table 1, Table 2, Tables S1 and S2). Section 4 reviews the recently published advances in the field of metabolome-oriented studies of starfish and sea cucumbers.

## 2. Overview of MS-Based Metabolomic Workflows in the Analysis of Starfish and Sea Cucumber Bioactive Compounds

**Figure 1:**
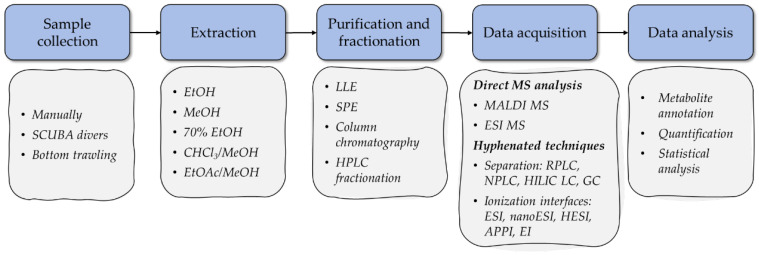
Main research stages in MS-based metabolomics studies of starfish and sea cucumber bioactive compounds.

In terms of workflow, a typical MS-based metabolomic study involves the stages of sample collection, extraction, fractionation and/or purification, measurement, identification, and analysis of the results (Figure 1). The analytical protocols used in MS-based marine metabolomics have several important differences from those used to analyze the metabolites of terrestrial animals and plants [41]. This section discusses the characteristic features of MS-based metabolomic approaches in the context of starfish and sea cucumber bioactive compounds, including steps of sample preparation, acquisition, and analysis.

### 2.1. Sample Preparation

The sample preparation stage, which comprises sample collection and extraction, is the most important in metabolomics research. Most of the studied starfish and sea cucumbers are collected from the coastal area manually or by SCUBA divers, or, if the depth exceeds 30 m, by bottom trawling. The main difficulties encountered in the collection of echinoderms are related to accessibility and their limited quantity. Many starfish and sea cucumbers are common species found in coastal areas where collection is unproblematic, while others occur in restricted or inaccessible geographic areas or in limited populations. Certain sea cucumber species, such as *Apostichopus japonicus* and *Holothuria scabra*, are aquaculture species, making their collection much simpler than the collection of wild specimens. In contrast to terrestrial ecosystems, when collecting marine samples the depth, salinity, and oxygen concentration of the water must be considered in addition to general factors such as temperature and light. A specimen’s location, physiological state, sex, and season can have a great metabolic influence. Difficulties are often caused by significant distances between the collection site and the laboratory, which requires more complicated logistics and specific sample preparation protocols.

Stress caused by handling induces responses in animals at the biochemical level [46] and can cause sea cucumber evisceration, the expulsion of the internal organs from the body [47]. To avoid such changes, as well as metabolomic changes resulting from enzymatic turnover during transportation or sample processing, it is highly recommended to quench the metabolism rapidly [48]. There are protocols designed for quenching, including flash-freezing using liquid nitrogen or dry ice, lyophilization, and freeze-drying; however, some of these are difficult to implement when animals are collected in the wild. Therefore, in most cases researchers use alternative protocols such as freezing or direct extraction with organic solvents [41].

The collected sample material must be processed to extract the metabolites of interest and remove salts and impurities. Extractions with organic solvents are commonly used for this purpose. Due to the high chemical diversity of metabolites, there is no single solvent capable of capturing all the required compounds without related impurities and contaminants. Generally, polar and semi-polar metabolites such as triterpene glycosides, asterosaponins, and gangliosides are preferentially extracted with hydro-alcoholic solutions, while lipid, sterol, terpene, and other non-polar compound extraction can be achieved with hydrophobic solvents (chloroform, hexane) or liquid–liquid extraction (LLE) by Folch’s [49] and Bligh and Dyer’s [50] methods. Extraction with methyl tert-butyl ether (MTBE) [51] can be used for the recovery of both polar and non-polar metabolites into separate fractions. In addition, the selected extraction protocols and solvents must be related to the analytical methods used.

It should be noted that most starfish and sea cucumber extracts contain significant amounts of salts, even if a non-polar solvent is used for extraction. Such samples are incompatible with analytical techniques such as mass spectrometry and NMR because of the effect of salts on analytical performance. For example, the presence of a small concentration of NaCl can cause the appearance of unexpected adducts at ESI MS, while larger concentrations can suppress analyte ionization and lead to salt crystal deposits in the ion source and the capillary, which can cause the instrument malfunction. MALDI MS is more tolerant to salt impurities and can be used for preliminary screening of extracts without additional purification. Desalting of marine extracts typically involves column chromatography (CC), liquid–liquid extraction, and solid-phase extraction (SPE).

Another problem can be the presence of lipid impurities and/or proteins in samples of polar secondary metabolites. For example, when extracting starfish polar steroid compounds or sea cucumber triterpene glycosides the crude hydro-alcoholic extracts may contain a large concentration of phospholipids, which can complicate chromatographic separations and suppress the ionization of the target analytes. If lipid compounds are not included in the target pool, additional purification of the extract can improve both LC separation and MS identification of target analytes. In order to remove such interfering compounds, column chromatography with Amberlite XAD-4, Sephadex LH-60, or other sorbents, LLE or SPE is usually used. In order to simplify the analysis of extremely complex extracts and obtain a mixture containing only structure-related metabolites of interest, fractionation using column chromatography, flash chromatography, or HPLC is used.

The choice of extraction solvent and purification methods affects the efficiency of the sample preparation stage. The use of unsuitable solvents and extraction methods can result in quantitatively and qualitatively incomplete extraction, while the use of suboptimal purification or fractionation procedures can lead to loss of the target metabolites. To the best of our knowledge, there are no published studies comparing the effectiveness of the most commonly used sample preparation protocols for the analysis of starfish and sea cucumber bioactive compounds.

### 2.2. Data Acquisition

Structural elucidation of starfish and sea cucumber bioactive compounds remains a difficult task due to the great diversity of these compounds and the complexity of the analyzed mixtures. Usually, these compounds form very complicated mixtures which are difficult to separate into pure compounds by chromatography. In the past, the application of chemical methods was required in order to identify the structure of such compounds. In particular, acid hydrolysis was used to recognize steroid and triterpene glycoside structures. While this approach allowed the partial characterization of aglycon structures and the determination of qualitative and quantitative monosaccharide composition, the destruction of native aglycon was frequent.

For a long time, electron ionization (EI) was the only possible mass spectrometry technique. Rashkes et al. carried out mass spectrometry research on six polyhydroxysteroid compounds and glycosides isolated from the Far Eastern starfish *Patiria pectinifera* and determined the characteristic fragmentation pattern of starfish polyhydroxysteroid under EI conditions [52]. EI and GC-EI MS were widely used for the determination of structures of aglycones and oligosaccharide chains of asterosaponins and triterpene glycosides after hydrolysis of glycosides and chemical derivatization of monosaccharides [20]. GC coupled with EI MS remains one of the most suitable metabolomic techniques for analyzing the wide range of volatile, semi-volatile non-polar compounds and derivatized polar metabolites. Electron impact ionization results in highly reproducible fragmentation patterns that can be used for identification by database search along with retention times indexes.

The application of fast atom bombardment (FAB) MS allows for analysis of the more polar and unstable compounds. Introduced in 1983, FAB has been successfully used for the determination of the structures of starfish steroid glycosides and sea cucumber triterpene glycosides as well as cerebrosides and gangliosides, which could not be analyzed by EI MS [53]. FAB mass spectra of starfish and sea cucumber glycosides can show molecular ions as well as fragmentation products, providing information about molecular formulae, the presence and location of sulfate groups, the structures of carbohydrate chains, and aglycon. Collision-induced dissociation (CID) experiments can provide additional structural information on the structural features of aglycon, the quantity and type of monosaccharides attached to aglycon, and their location.

Electrospray ionization (ESI) and Matrix-Assisted Laser Desorption/Ionization (MALDI) have significantly expanded the possibilities of mass spectrometry for the analysis of natural products. ESI has had an enormous impact on the analysis of polar and non-volatile molecules as well as large biomolecules. In contrast to electron ionization, in-source fragmentation under ESI conditions is practically unrealized; tandem MS methods are used to initiate the fragmentation of these ions. ESI mass spectrometry is currently the most common ionization technique; it has been widely used for the characterization of natural compounds, including steroid and triterpene glycosides, polar lipids, and other compounds from purified starfish and sea cucumber extracts. MALDI MS is another efficient method for the analysis of natural compounds. The necessity of using matrices and the presence of matrix ion peaks at spectra in the low mass range are drawbacks; however, due to its high sensitivity, high speed of analysis, and tolerance to inorganic salts impurities, MALDI MS is widely used for rapid screening and chemical characterization of complex mixtures. Recent advances in analytical techniques, including high-resolution time-of-flight (TOF), Fourier transform (FT), and Orbitrap mass analyzers have high scan speeds along with extended dynamic range and sensitivity, allowing for the development of hybrid instruments and new ionization interfaces such as nanoelectrospray (nanoESI) and heated electrospray ionization (HESI) and leading to the establishment of high-throughput protocols for the analysis of the most complex mixtures of natural compounds. The development of hyphenated techniques combining liquid chromatography or gas chromatography with mass spectrometry (LC-MS or GC-MS) makes allows for straightforward analysis of the compounds present in complicated extracts.

### 2.3. Data Analysis

Data analysis is the next important stage of MS-based research. Generally, the processing of data obtained using chromatography-MS methods has included the steps of identifying *m*/*z* signals, chromatographic peak detection, filtering, alignment, and identification [54]. Many freely available (XCMS [55], MZmine 2 [56], OpenMS [57], and MS-DIAL [58]) and commercial software tools are currently available for the processing of LC-MS and GC-MS data. While the processing of GC-MS data is well-established and relatively simple, the results are usually limited to known compounds presented in databases. LC-MS is a more versatile method, covering broad chemistries and sensitivity ranges, although it produces more complex data. Due to the lower resolution and reproducibility of LC separation and the presence of adduct, isotope, fragment, and contamination peaks in ESI spectra, LC-MS data processing is much more difficult.

In certain cases, special approaches are useful for data processing. In order to process MS profiling data, methods based on scanning neutral losses, characteristic fragments, and an in-house library for rapid screening of the compounds of interest are often used. For example, the construction of ion chromatograms for negative fragment ions at *m*/*z* 96.96 can be used for detecting sulfated compounds like asterosaponins and sulfated triterpene glycosides, and cerebrosides can be detected according to the neutral loss fragments of 180 Da [59].

Similar to common metabolomic studies, metabolite identification is a current bottleneck in the analysis of starfish and sea cucumber metabolites. Chromatography-MS-based analysis can result in a huge number of peaks that are extremely difficult to identify. Even when analyzing well-studied organisms, only small percentages of the data collected in a typical LC-MS experiment can be matched to known molecules [60]. The chemical composition of starfish and sea cucumbers remains poorly investigated and the percentage of identified compounds can be extremely low. According to Metabolomics Standards Initiative recommendations, high identification confidence can be obtained by comparing an accurate high-resolution monoisotopic mass, MS/MS spectra, and retention times with data from an authentic chemical standard [61]. However, the available libraries of certified standards do not cover the entire scope of biochemical diversity, especially in the area of marine bioactive compounds. Although only putative annotation is possible without matching experimental data to data for authentic chemical standards [61], the availability of comprehensive open-access databases is extremely important for the successful application of mass spectrometry to the analysis of complex mixtures of natural compounds. Existing databases cover various natural compounds [62], and several databases, such as the GNPS database [63], MassBank [64], Metlin [65], the Human Metabolome Database [66], and MassBank of North America (https://mona.fiehnlab.ucdavis.edu, accessed on 1 April 2022) include MS/MS spectra of natural compounds from different sources. Databases such as the Dictionary of Marine Natural Products (https://dmnp.chemnetbase.com, accessed on 1 April 2022) and MarinLit (https://marinlit.rsc.org, accessed on 1 April 2022) include structural information, and the NMR and UV spectra of marine-derived compounds, although MS and MS/MS data on marine natural compounds in all existing databases is extremely limited. There are currently no databases covering taxonomic, structural, and experimental mass spectrometry data on bioactive metabolites of marine echinoderms.

Moreover, unlike peptides, oligosaccharides, and lipids, the MS fragmentation of most secondary metabolites is less studied due to the vast structural variability, and the de novo identification of metabolites by MS/MS spectra is very difficult. Several computational approaches based on machine learning or quantum chemistry calculations have been proposed for the in silico generation of MS/MS spectra or the prediction of structural features of compounds based on the experimental MS/MS spectra [67,68]. The molecular networking approach is based on the clustering of detected compounds by the similarity of their MS/MS spectra and allows for the annotation of related metabolites [63]. Using models for the in silico prediction of LC retention times can help to improve the reliability of identification in metabolomics analysis [69]. However, despite the recent advances in computational approaches, the currently used algorithms need to be significantly improved before effective identification and structural elucidation of marine bioactive compounds is possible. Along with the huge degree of structural variability, these issues limit the application of MS for annotation, dereplication, and structural elucidation in studies of metabolites from marine organisms.

In the case of research aimed at discovering metabolomic alterations between distinct biological groups of organisms, the statistical analysis is applied to the peak lists obtained after the processing of MS data. The choice of statistical methods is often determined by the study design, and can be divided into univariate (*t*-test, analysis of variance (ANOVA), fold-change analysis) and multivariate (unsupervised Principal Component Analysis (PCA) and supervised Partial Least Squares Discriminant Analysis (PLS-DA)) methods of analysis. Generally, the statistical methods used in the analysis of starfish and sea cucumber metabolites are the same as those used for conventional metabolomics studies, which are thoroughly discussed in [70]. Tables S1–S3 provide a general overview of the statistical approaches used for the treatment of analytical data in research on starfish and sea cucumber bioactive compounds.

## 3. MS-Based Metabolomic Profiling Approaches to the Study of Starfish and Sea Cucumber Bioactive Compounds

### 3.1. Starfish Polar Steroid Compounds

**Figure 2:**
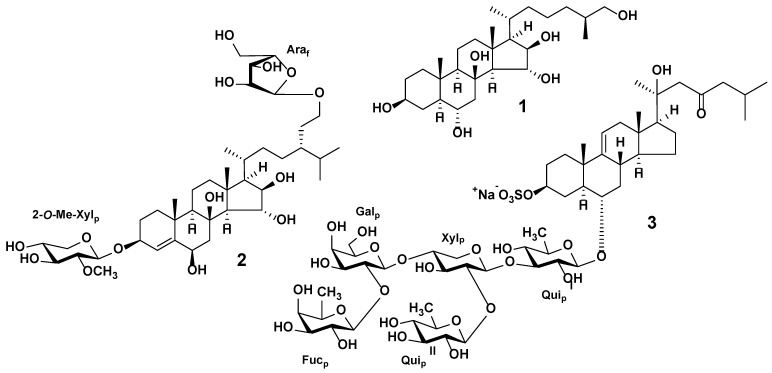
The structures of typical starfish polyhydroxysteroid (5α-cholestane-3β,6α,8,15α,16β,26-hexaol (**1**) from the starfish *Protoreaster nodosus* [71]), a glycoside of polyhydroxysteroid (linckoside A (**2**) from the starfish *Linckia laevigata* [72]), and asterosaponin (thornasteroside A (**3**) from the starfish *Acanthaster planci* [73]).

Unlike other echinoderms, starfish are characterized by a wide variety of steroid compounds, both non-polar sterols and polar steroid compounds. The latter form a large group of biologically active compounds, including polyhydroxysteroids, related glycosides, and steroid oligoglycosides (asterosaponins) (Figure 2). Starfish polyhydroxysteroids are steroid compounds, and usually contain from four to nine hydroxy groups in a steroidal nucleus and side chain. Polyhydroxylated glycosides have one, two, or rarely three monosaccharides attached to a steroid moiety, either to side chains or to the steroid nucleus and side chain simultaneously. The most common sugar residues in these compounds are xylose or its derivatives and arabinose. Polyhydroxylated glycosides have been found in both sulfated and non-sulfated forms. A characteristic feature of the asterosaponins is the 3β,6α-dihydroxysteroid aglycon with a 9(11)-double bond and a sulfate group at C-3. The asterosaponin carbohydrate chain consists of four to six sugars and is attached to C-6. The oligosaccharide chain of pentaosides contains one branching at the second monosaccharide, while hexaosides can have one or two branches at the second and third monosaccharide residues of the chain. Hexoses (glucose, galactose), pentoses (arabinose, xylose), and deoxyhexoses (fucose, quinovose) are the most common sugar residues in asterosaponins. Monosaccharides in asterosaponins are always in pyranose forms and are connected, as a rule, by β-glycosidic bonds (Figure 2).

Demonstrating significant structural diversity, individual representatives of starfish polar steroids show a variety of biological effects including cytotoxic, neuritogenic hemolytic, antibacterial, antiviral, and anti-inflammatory effects [12,20,27,28,29,30,31]. Several starfish polar steroids are promising antitumor and cancer-preventing agents [14]. A recent study has reported that starfish polar steroids in combination with X-ray radiation affect colony formation and apoptosis induction in human colorectal carcinoma cells [74]. Starfish polar steroids have shown a combined anticancer effect with alga polysaccharides on human cell lines in models of 2D and 3D cultures [75,76].

ESI and MALDI are currently the most suitable and widely used ionization techniques for analyzing starfish polar steroids (Table 1). Typically, starfish sulfated steroid glycosides are detected as [M + Na]^+^ and [M − Na]^−^ ions in the positive and negative ion modes of ESI MS, respectively. Non-sulfated polyhydroxysteroids and related glycosides are usually revealed as [M − H]^−^ and [M + Cl]^−^ ions in the negative ion mode and as [M + Na]^+^ ions in the positive ion mode. Although both positive and negative ion mass spectra are good enough to characterize all types of glycosides, the negative ion mode contains peaks of higher intensities and is more suitable for the analysis of sulfated compounds, while non-sulfated glycosides are analyzed by the positive ion mode [77,78,79].

**Figure 3:**
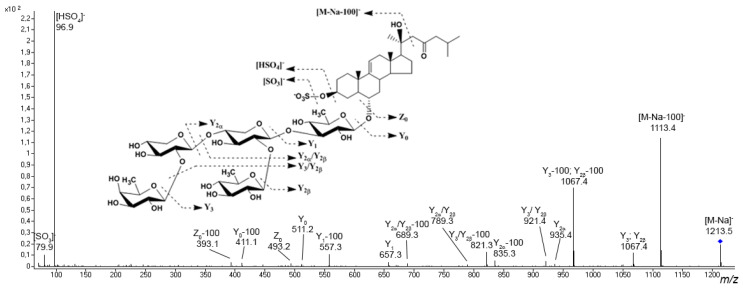
ESI MS/MS spectrum of [M − Na]^−^ precursor ion at *m*/*z* 1213 identified as ophidianoside F (modified from [77]).

Preliminary asterosaponin structures can be predicted from experimental tandem MS data because typical fragment ions and neutral losses provides valuable information about aglycon structures and sequences of monosaccharide units in carbohydrate chains. Tandem mass spectra show intensive characteristic fragment peaks for all asterosaponins and sulfated glycosides, indicating the sulfate group (peak at *m*/*z* 96.9 in negative ion mode and neutral loss of 120 Da and peak at *m*/*z* 142.9 in positive ion mode) (Figure 3). In the negative product ion spectra, an intense Y-type ion series (nomenclature by Domon and Costello [80]) associated with the cleavages of glycosidic bonds and corresponding sequential losses of sugar units has been observed. The analogous Y-type product ion series corresponding to losses of monosaccharide units as well as B- and C-type product ion series can be observed in the positive ion spectra of asterosaponins. The fragmentation of certain asterosaponins under CID conditions produces a very intense characteristic product ion series corresponding to the loss of side chain neutral fragments. For example, the spectra of many asterosaponins display neutral loss of a fragment of 100 Da as well as the Y–100 product ion series. This fragmentation corresponds to the loss of the C_6_H_12_O molecule associated with the C-20–C-22 bond cleavage and 1H transfer, which is characteristic of asterosaponins containing an aglycon with a 20-hydroxy-cholestan-23-one side chain (Figure 3) [20]. The similar product ion series Y–114 and Y–128 indicate aglycons with 20-hydroxy-24-methyl-cholestan-23-one and 20-hydroxy-24-ethyl-cholestan-23-one side chains.

The structural characterization of polyhydroxysteroids and glycosides of polyhydroxysteroids is challenging due to the great diversity of compounds in this class. Spectra of polyhydroxysteroid compounds and related glycosides contain fragmentation patterns indicate a number of hydroxy groups and structures of the side chains and steroid nuclei. Tandem spectra of polyhydroxysteroid glycosides with sulfated monosaccharide unit usually show diagnostic ion B_0_ at *m*/*z* 241.0 [C_6_H_9_O_8_S]^−^, 225.0 [C_6_H_9_O_7_S]^−^ or 210.9 [C_5_H_7_O_7_S]^−^, which are characteristic of sulfated hexose, methylated pentose, or pentose units, respectively, whereas the presence of intense Y-type ions is associated with non-sulfated compounds or sulfated aglycon. In certain cases the A- and X-type product ions formed by cross-ring cleavages of sulfated monosaccharides can be detected, potentially allowing isomeric monosaccharides with different positions of the sulfate group to be distinguished [81,82]. In other cases, the structure of the polyhydroxysteroidal aglycon can be proposed from both obtained MS data and from biosynthetic considerations [77,83].

Table 1 provides a general overview of the approaches used for metabolomic profiling of starfish polar steroids and triterpene glycosides of sea cucumbers, including extractions, purification methods, and analytical techniques. Table S1 provides more expansive technical details of these particular approaches, including sample preparation protocols and the instrumental setups of the ESI MS, MALDI MS, and hyphenated techniques.

**Table 1:** Selected examples illustrating MS-based approaches for the analysis of starfish polar steroids and sea cucumber triterpene glycosides *.

Species Name	Extraction	Purification Methods	MS Approach	Research Results	Number of Detected Analytes	Ref.
Asteroidea						
*Asterias rubens*	MSPD extraction		RPLC-NMR-ESI-IT MS	A combination of MSPD extraction with on-flow LC–NMR–MS for rapid chemical screening and structural elucidation was applied; a series of new asterosaponins were found and their structures were established	17 asterosaponins	[84]
*A. rubens*	90% MeOH	LLE, CC	MALDI-QTOF MS; MALDI-TOF/TOF MSI; RPLC-ESI-QQQ MS	A series of known and new asterosaponins were detected and characterized; localization, inter- and intra-organ variability of asterosaponin were described	26 asterosaponins	[85,86]
*Aphelasterias japonica*	EtOH	SPE	RPLC-ESI-QTOF MS	A series of new polar steroid compounds were detected and characterized; a theoretical scheme of biogenesis of several polar steroids was proposed	33 asterosaponins, 28 polyhydroxylated glycosides, 7 polyhydroxysteroids	[77]
*Patiria pectinifera*	EtOH	SPE	RPLC-ESI-QTOF MS	A series of new polar steroid compounds were detected and characterized; peculiarities of the biosynthesis of the starfish polar steroids were discussed. Changes in steroid metabolome induced by environmental factors were studied	35 asterosaponins, 22 polyhydroxysteroids, and 15 polyhydroxylated glycosides	[78,87]
*Luidia senegalensis*	70% EtOH	SPE	RPLC-ESI-IT MS	New asterosaponins were detected and annotated	5 asterosaponins, 2 polyhydroxysteroids	[88]
*Lethasterias fusca*	EtOH	LLE, SPE	nanoRPLC-CSI-QTOF MS	A series of new polar steroids compounds were detected and their fragmentation behaviors were extensively investigated; variations in the distribution of individual representatives in different organs were found	106 asterosaponins, 81 polyhydroxylated glycosides, 14 polyhydroxysteroids	[79,89]
*Echinaster sepositus*	60% MeOH	LLE	ESI-QOrbitrap MS	New asterosaponins were detected and annotated; significant inter-organ variability in asterosaponins was demonstrated	11 asterosaponins	[90]
*Heliaster helianthus*	EtOH	LLE, CC	ESI-QTOF MS	The presence of sulfated steroidal glycosides in the fractions studied was confirmed and their structures were established	1 asterosaponin, 2 polyhydroxylated glycosides	[91]
Holothuroidea						
*Holothuria forskali*	70% EtOH	LLE, CC	MALDI-QTOF MS; RPLC-ESI-QTOF MS	A series of triterpene glycosides were detected and characterized; variations in triterpene glycoside composition in Cuvierian tubules and body walls were demonstrated	26 triterpene glycosides	[92]
*H. forskali*	70% EtOH	LLE, CC	MALDI-TOF/TOF MS; MALDI-TOF/TOF MSI	Statistical differences in triterpene glycoside distribution between control and stressed groups were described	8 triterpene glycosides	[93]
*H. atra*, *H. leucospilota*, *Pearsonothuria graeffei*, *Actinopyga echinites*, *Bohadschia subrubra*	70% EtOH	LLE, CC	MALDI-QTOF MS; RPLC-ESI-QTOF MS	A series of new and known glycosides were detected and characterized; variations between species and between body compartments were established	*H. atra*—4, *H. leucospilota*—6, *P. graeffei*—8, *A. echinites*—10, *B. subrubra*—19 triterpene glycosides	[94]
*H. forskali*	70% EtOH	LLE, CC	MALDI-QTOF MS; RPLC-ESI-QTOF MS	Localization of triterpene glycosides in the body wall tissues was described; variations of secreted glycosides were found in the seawater surroundings of non-stressed and stressed animals	8 triterpene glycosides	[95]
*H. scabra*, *H. impatiens*, *H. fuscocinerea*	70% EtOH	LLE, HPLC	nanoRPLC-ESI-QTOF MS; MALDI-FTICR MS	Triterpene glycoside compositions of three sea cucumber species were described; variations and sample-specific compounds were found	*H. scabra*—32, *H. impatiens*—32, *H. fuscocinerea*—33 triterpene glycosides	[96]
*H. scabra*	MeOH	LLE, CC	MALDI-QTOF MS	The triterpene glycoside composition of the *H. scabra* body wall was characterized, as was processed holothurian,	6 triterpene glycosides	[97]
*H. sanctori*	MeOH	LLE, CC	MALDI-QTOF MS	Qualitative and quantitative differences in the body wall and Cuvierian tubules of composition were described	18 triterpene glycosides	[98]
*Eupentacta fraudatrix*	EtOH	SPE	RPLC-ESI-QTOF MS	A series of triterpene glycosides were discovered and characterized; qualitative and quantitative variations in the body wall and viscera were found	54 triterpene glycosides	[99]
*H. scabra*		SPE	RPLC-multimode source-QTOF MS	Several known and new triterpene glycosides were identified in conditioned water of *H. scabra*	16 triterpene glycosides	[100]
*H. forskali*	MeOH	LLE	MALDI-QTOF MS; RPLC-ESI-QQQ MS; RPLC-ESI-IM-QTOF MS	The triterpene glycoside compositions of the body wall, gonads, and Cuvierian tubules of *H. forskali* were described	26 triterpene glycosides	[101]
*H. leucospilota*	70% EtOH; H_2_O or *n*-BuOH	LLE	MALDI-TOF/TOF MS; MALDI-TOF/TOF MSI	The presence of triterpene glycosides was confirmed in the body wall and epidermis extracts; epidermal pigmented cells were reported to involve in the accumulation and release of the triterpene glycosides to the surrounding seawater	12 triterpene glycosides	[102]
*H. atra*	EtOAc/MeOH	LLE	RPLC-ESI-QOrbitrap MS	A combination of LC-MS profiling and molecular networking followed by target compound isolation was applied; variations in triterpene glycoside composition between *H. atra* from the Persian Gulf and previously reported results were described	15 triterpene glycosides (4—isolated as pure compounds)	[103]
*Apostichopus japonicus*	70% EtOH	LLE	RPLC-ESI-QOrbitrap MS	Variability in triterpene glycoside composition among different types of *A. japonicus* was described	5 triterpene glycosides	[104]
*H. polii*, *H. leucospilota*, *H. atra*, *H. edulis*, *Bohadschia marmorata*, *Actinopyga mauritiana*	96% EtOH	LLE	RPLC-ESI-QOrbitrap MS	MS-based profiling results were applied for chemotaxonomy of sea cucumber species	4 triterpene glycosides; 15 fatty acids, 45 triacylglycerols	[105]
*H. whitmaei*, *H. hilla*, *H. atra*, *H. edulis*, *Bohadschia argus*, *B. vittiensis*, *Bohadschia* sp., *Actinopyga echinites*, *A. mauritiana*	MeOH:EtOAc, MeOH	LLE, SPE, HPLC	RPLC-ESI-QTOF MS	A series of triterpene glycosides were detected in crude extracts; anti-fouling activity of sea cucumber extracts was found to be species-specific and related to total concentration of triterpene glycosides.	102 triterpene glycosides in crude extracts (including 23 triterpene glycosides in *B. argus* fractions)	[106]
*H. scabra*	MeOH	flash chromatography, LLE	MALDI-QTOF MS; RPLC-ESI-IM-QTOF MS	The qualitative and quantitative composition of triterpene glycosides in dried viscera and its desulfation by microwave activation products were described	26 triterpene glycosides	[107]

* Abbreviations: CC, column chromatography; CSI, captive spray ionization; ESI, electrospray ionization; FTICR, Fourier-transform ion cyclotron resonance; HPLC, high-performance liquid chromatography; IM, ion mobility; IT, ion trap; LC, liquid chromatography; LLE, liquid-liquid extraction; NMR, nuclear magnetic resonance; MALDI, matrix-assisted laser desorption/ionization; MS, mass spectrometry; MSI, mass spectrometry imaging; MSPD, matrix solid-phase dispersion; nanoESI, nanoelectrospray; QOrbitrap, quadrupole-Orbitrap; QTOF, quadrupole time-of-flight; QQQ, triple-quadrupole; RPLC, reverse-phase liquid chromatography; SPE, solid-phase extraction; TOF, time-of-flight.

Polar steroid compounds are usually extracted from animal materials using methanol, ethanol, or hydro-alcoholic solutions. As crude extracts contain a large concentration of impurities and inorganic salt, there is a need for additional purification procedures before analysis. SPE with reverse-phase (RP) sorbents is the most fast and versatile way of obtaining the purified total fraction of polar steroids [77,78]. Regarding asterosaponin fraction extraction, LLE is preferred. The most commonly used protocol is adapted from that in [108], and includes the dilution of the dry extract in 90% methanol followed by successive partitioning against *n*-hexane, dichloromethane, and chloroform. After column chromatography with an Amberlite XAD-4 column, the eluate is extracted against isobutanol. The resulting fraction contained purified asterosaponins [85,90].

Although LC coupled to MS through electrospray ionization interface is the most popular combination for profiling due to its efficiency, versatility, and capability in analyzing isomeric compounds, MALDI is commonly used as the primary technique for the rapid screening of extracts [85]. All of the researchers reviewed here used columns with RP C18 sorbents for analytical separation; however, it should be noted that the high complexity of the extracts and fractions places high demands on both chromatography and detection. The use of high-resolution MS analyzers (TOF and Orbitrap) enables calculation of the elemental composition of the analytes via high accuracy measurements and true isotopic patterns, while using tandem techniques allows for structural characterization of the detected compound.

LC-NMR-MS was used for the profiling and characterization of *Asterias rubens* asterosaponins [84,109]. The on-flow LC-NMR-MS screening showed novel asterosaponins in *A. rubens*, and their tentative structures were proposed by MS and NMR data [109]. Using a similar experimental setup, the authors replaced time-consuming classical extraction with matrix solid-phase dispersion (MSPD) extraction, which combines both sample homogenization and extraction in a single step starting from the intact sample material. The structures of seventeen asterosaponins were established based on complementary structural information from both MS and NMR detection, including 1H-NMR spectra obtained in on-flow mode, 2D WET-TOCSY spectra from the MS-triggered stopped-flow mode, information about molecular mass before and after H-D exchange, and fragmentation patterns and characteristic neutral losses [84].

Further studies on *A. rubens* using a combination of MALDI MS, MALDI imaging (MALDI MSI), and LC-MS have focused on the diversity, body distribution, and localization of asterosaponins [85,86]. Asterosaponins from the body walls, stomach, pyloric caeca, and gonads were extracted and analyzed by MALDI-TOF MS and LC-ESI MS [85]. As a result, seventeen known and nine novel asterosaponins were detected. It was found that each organ was characterized by a specific mixture of asterosaponins, and that their concentration varies considerably among individuals. MALDI MSI was used to clarify the inter- and intraorgan distribution of asterosaponins [86]. Sample preparation is a particularly important step in MALDI imaging. Because the starfish body wall contains calcareous ossicles, the researchers used carboxymethyl cellulose as an embedding medium to facilitate the cryosectioning procedure. The results confirmed that asterosaponin distributions are not homogeneous, and revealed that certain asterosaponins are located both inside the body wall and within the outer mucus layer, where they probably protect the animal.

As a part of starfish polar steroid exploration, metabolite profiling of polar steroids in the Far Eastern starfishes *Aphelasterias japonica* and *Patiria pectinifera* was performed [77,78]. A detailed LC-MS analysis of the complicated mixture of polar steroids from *A. japonica* revealed 68 polar steroid metabolites, including 33 asterosaponins, 28 polyhydroxysteroid glycosides, and seven polyhydroxysteroids [77]. Fragmentation analysis indicated asterosaponins with rare and atypical units in their oligosaccharide chains that have thus far not been identified from marine sources. The profiling of polar steroid compounds of *P. pectinifera* using the LC-MS allowed many different polar steroid compounds to be discovered [78]. LC-ESI MS analysis revealed 72 components (35 asterosaponins, 15 sulfated glycosides of polyhydroxysteroids, and 22 polyhydroxylated steroids). Annotation was based on MS data obtained in both negative and positive ion modes. Liquid chromatography coupled with atmospheric pressure photoionization (LC-APPI) MS was applied for non-sulfated polyhydroxysteroid compounds. APPI MS/MS exhibited extensive fragmentation, with sequential neutral losses of H_2_O molecules and cleavages in side chains and tetracyclic nucleus. The comparison of the steroid constituents of *P. pectinifera* and *A. japonica* revealed significant differences associated with details of the biosynthesis of starfish polar steroids.

A combination of SPE and ultra-high performance liquid chromatography (UPLC) coupled with ion trap (IT) mass spectrometry was used to profile asterosaponins from the Brazilian starfish *Luidia senegalensis* [88]. Seven components were detected as a result, and five of which were characterized as asterosaponins. ESI MS was used together with NMR to detect and characterize asterosaponins and sulfated polyhydroxysteroid glycosides in bioactive fractions obtained by LLE and chromatography purification of the ethanolic extract from the starfish *Heliaster helianthus* [91].

The profiling of polar steroids from the starfish *Lethasterias fusca* was carried out by nanoflow liquid chromatography coupled with captive spray ionization (CSI) mass spectrometry [79]. As a result, the structure of the largest number of polar steroid metabolites was discovered, and the MS fragmentation of a large series of starfish polar steroids was studied. A total of 207 compounds, including 106 asterosaponins, 81 glycosides of polyhydroxysteroids, and 14 polyhydroxylated steroids, were detected and characterized. Further study of the distribution of the detected compounds in *L. fusca* body components showed that the polar steroid compositions in the body walls, coelomic fluid, gonads, stomach, and pyloric caeca were qualitatively and quantitatively different [89]. Research on the distribution of asterosaponins from *Echinaster sepositus* revealed eleven compounds, and found significant variability in asterosaponin composition depending on the organ, sex, and season [90].

In summarizing the aforementioned results it is necessary to note the huge variety of starfish polar steroids. Each studied species contained dozens, and in several cases, hundreds of polar steroids, most of which had not been previously described [77,78,79]. Additional studies of the polar steroids in the studied species often lead to the identification of both known compounds and new previously-undescribed metabolites. For example, the study of asterosaponins of *A. rubens* by LC-NMR-MS revealed seventeen asterosaponins [84]. Subsequent studies led to the detection of both previously discovered compounds as well as the discovery of new asterosaponins [85,86]. This is related both to the instrumental advancements involving increasing sensitivity and selectivity of analysis and to the great variability in the polar steroid composition of starfish, even among representatives of the same species. The observed large structural variability together with the huge number of structures in each species studied and the small number of species studied (a total of six species have been studied using metabolomic methods) may indicate a potentially huge chemical space for starfish polar steroids.

Most studies have focused on both the description of the structural diversity and on the study of the localization of the discovered compounds. It has been determined that each organ is characterized by a certain composition of polar steroids. The comparison of the content of individual steroids in different starfish organs probably suggests the different biological roles of these metabolites in the starfish. Asterosaponins, which are the most toxic starfish compounds, have been found in all organs of the starfish [85,86,89]. However, the body walls often show the highest content of asterosaponins. In addition, these compounds have been found in the outer layer of mucus [86]. This may be due to the toxic, protective, or antimicrobial properties of these compounds. The main potion of polyhydroxysteroid glycosides is located in the pyloric caeca, which confirms the digestive function of these steroids in starfish [89,110]. At the same time, the levels of polar steroids can vary greatly depending on the individual, season, and sex [85,90,111]. This high inter-individual variability may be associated with different physiological statuses of the animals, and partly with the biogenesis of certain compounds from dietary steroids.

### 3.2. Sea Cucumber Triterpene Glycosides

**Figure 4:**
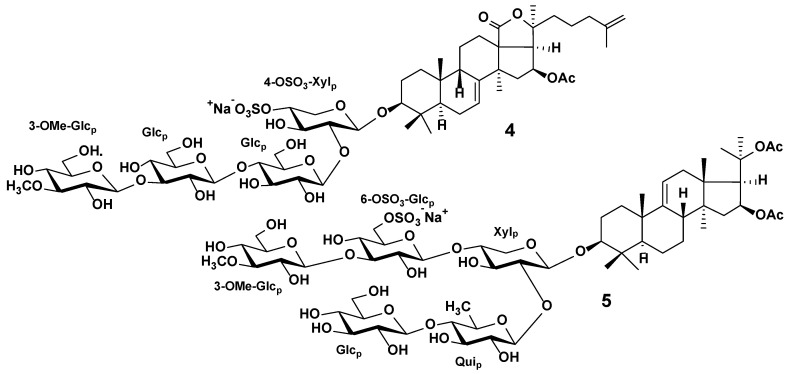
The structures of typical holothurian triterpene glycosides with holostane aglycon (okhotoside B_1_ (**4**) from the sea cucumber *Cucumaria okhotensis* [112]) and rare non-holostane aglycon (kurilosides A_1_ (**5**) from the sea cucumber *Thyonidium* (*=Duasmodactyla*) *kurilensis* [113]), demonstrating different carbohydrate chain architecture.

Triterpene glycosides are the characteristic secondary metabolites of sea cucumbers. Their chemical structures are characterized by the large variability of certain structural features, although the general structure of these compounds is rather conservative. Most sea cucumber triterpene glycosides have a lanostane-type aglycon with an 18(20)-lactone. Usually, aglycon has a polycyclic nucleus with a 7(8)- or 9(11)-double bond and oxygen-containing substituents, which may be bonded to C-12, C-17, or C-16. Structures of the side chains of aglycons demonstrate significant natural diversity and may have one or more double bonds, hydroxyl or acetate groups, and other substituents. Certain glycosides have aglycons with shortened side chains. The carbohydrate chains of sea cucumber glycosides may include up to six sugar units and be attached to C-3 of the aglycon. Xylose, glucose, quinovose, 3-*O*-methylglucose, and rarely 3-*O*-methylxylose are the most common sugar residues in triterpene glycosides. The first monosaccharide unit is always xylose, and monosaccharides with the 3-*O*-methyl group are always terminal ones. Many glycosides have up to four sulfate groups in the first xylose, glucose, and 3-*O*-methylglucose units. The oligosaccharide chains that have up to four monosaccharide units usually represent a linear structure, while the penta- and hexaosides contain a branching at the first or second monosaccharide unit (Figure 4) [21,23,24,32,33].

Triterpene glycosides demonstrate both biological and pharmacological effects, including cytotoxic, antifungal, bactericidal, antiviral, and antiparasitic effects [21,22,32,33]. The most interesting of these is the ability of certain glycosides to induce apoptosis and inhibit tumor cell growth; thus, sea cucumbers have become a promising source for the discovery of new drugs [114]. Additionally, certain triterpene glycosides have been reported to exhibit immunomodulatory properties [115]. Several species of sea cucumber are an important aquaculture resource and are used as functional foods [116]. It has been suggested that dietary triterpene glycoside supplements can improve lipid metabolism, significantly suppress adipose accumulation, and reduce serum and hepatic lipids [117].

**Figure 5:**
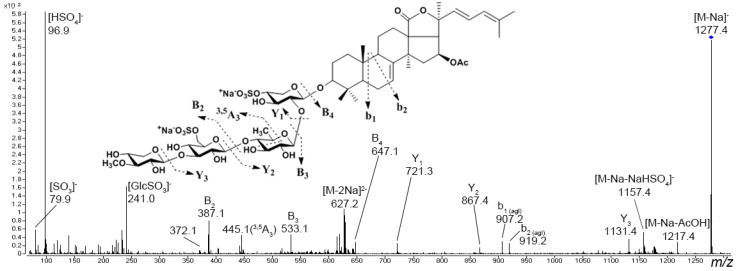
ESI MS/MS spectrum of [M − Na]^–^ precursor ion at *m*/*z* 1277, identified as cucumarioside F_2_ (modified from [118]).

Most triterpene glycosides have been detected within *m*/*z* range from 1000 to 1600 as [M + Na]^+^ ions. In most cases, sulfated and disulfated triterpene glycosides are detected in negative ion mode as [M − Na]^−^ and [M − 2Na]^2−^ ions, respectively, whereas non-sulfated compounds are detected as [M − H]^−^ ions. The tandem mass spectra of triterpene glycosides usually reveal B- and C-type product ion series arising from the cleavage of glycosidic bonds. These product ion series are characteristic, and they provide information about the sequence of monosaccharide residues in carbohydrate chains (Figure 5).

Tandem mass spectra of many triterpene glycosides show typical mass losses related to aglycon fragmentation, and can provide information about the structure of the nucleus and the side chain. In the MS/MS spectra of certain glycosides a mass loss of 60 Da between the precursor and the intense fragment ion has been detected, which corresponds to the loss of the C_2_H_4_O_2_ molecule and is a characteristic of glycosides containing an acetoxy group. An intense fragment ion with a mass loss of 104 Da from the precursor is related to the loss of a [C_2_H_4_O_2_ + CO_2_] fragment, which is characteristic of compounds with an acetoxy group and an 18(20)-lactone cycle. Certain triterpene glycosides tend to lose the neutral fragments of the side chain under CID conditions.

Regarding the extraction of triterpene glycosides from animal tissue, the most preferred solvents are 70% ethanol and methanol, although ethanol and ethyl acetate:methanol mixtures have been used (Table 1 and Table S1). Crude extracts must be purified before analysis to remove inorganic salts, lipid, and protein contaminants. For these purposes, most researchers use successive liquid–liquid partitioning against *n*-hexane, dichloromethane, and chloroform, followed by column chromatography with an Amberlite XAD-4 column and extraction against butanol or similar LLE-based protocols. Another purification approach includes SPE with C18 cartridges [99,100]. Omran et al. used LLE with a solvent combination of MTBE/MeOH/H_2_O for the purification of crude ethanol extract to obtain the polar fraction of the triterpene glycosides and non-polar fraction of lipids in a single extraction step [105]. The purified extracts can be fractionated using HPLC [96,106] or flash chromatography [107].

MALDI MS is often used as the primary technique for rapid screening of triterpene glycoside mixtures. As sea cucumber triterpene glycosides are characterized by the presence of isomeric compounds, the LC-MS technique can be used as a tool for discriminating different isomers and structure confirmation. In order to separate glycosides, most LC-MS applications use analytical columns with C18 sorbents. Ion mobility technology provides additional orthogonal separation for the discrimination and structural characterization of isomeric compounds [119].

A combination of MALDI MS and ESI MS was used to annotate the triterpene glycosides in purified fractions of the Australian sea cucumber *Holothuria lessoni* [120,121,122,123]. As a result, a series of known and novel triterpene glycosides were annotated by extensive MS fragmentation. It should be noted that the authors determined the structures of novel glycosides based only on MS data. However, it is known that different epimeric monosaccharides as well as types of bonds between sugars and absolute configuration of asymmetric atoms cannot be strictly distinguished by MS [124]. The proposed structures are therefore tentative, and must be verified by additional approaches such as NMR spectroscopy.

MS-based approaches were used for screening, characterization, and study of the bodily distribution of the triterpene glycosides of the sea cucumber *Holothuria forskali* [92,93,95]. Triterpene glycosides were extracted from two different body components, the body wall and the Cuvierian tubules (a defensive organ that can be ejected in response to predator attacks), and analyzed by a combination of MALDI MS, MALDI MSI, and LC-ESI MS. The analysis revealed at least 26 triterpene glycosides, including twelve glycosides in the body wall and twenty-six in the Cuvierian tubules. The glycosides detected in the body wall were found in the Cuvierian tubules, with the latter containing fourteen other specific glycosides as well. A more detailed study of triterpene glycoside localization in the body wall revealed that the glycosides were mainly localized in the epidermis and mesothelium [95]. A combination of MALDI, LC-ESI MS, and LC-ESI-IM MS was used in order to better tackle the structural complexity of *H. forskali* glycosides [101]. As a result, at least 10, 16, and 22 different triterpene glycosides within the body wall, gonads, and Cuvierian tubules, respectively, were detected. Glycosides with pentasaccharide chains were dominant within the extracts from the gonads and the Cuvierian tubules, whereas the body wall extract exhibited equally abundant tetra-, penta- and hexaosides. In addition, the authors described the interaction of branched triterpene glycosides with sodium ions, and proposed a new schematic for data representation using sector diagrams constructed from MS data.

The diversity of triterpene glycosides was studied in five tropical sea cucumber species [94]. Triterpene glycosides from the body wall and the Cuvierian tubules were extracted and analyzed with a combination of MALDI MS, ESI MS, and LC-MS. The researchers indicated that the smallest number of glycosides was observed in *Holothuria atra*, which contained a total of four compounds, followed by *Holothuria leucospilota*, *Pearsonothuria graeffei*, and *Actinopyga echinites* with six, eight, and ten compounds, respectively. *Bohadschia subrubra* showed the highest triterpene glycoside diversity. Differences between the glycoside composition in the body walls and the Cuvierian tubules were highlighted.

The profiles of three tropical sea cucumber species were determined and compared to examine their chemical diversity with phylogenetic data [96]. Semi-purified extracts from the body wall of *Holothuria scabra*, *H. impatiens*, and *H. fuscocinerea* were first analyzed by MALDI-FT MS and chip-HPLC-ESI MS. The obtained data showed holothurines common for three species (for example, holothurin A) as well as glycosides specific for certain species (for example, impatienside A in *H. impatiens*). Glycosidic fractions of three species contained approximately the same number of compounds (32 glycosides in *H. scabra* and *H. impatiens* and 33 glycosides in *H. fuscocinerea*); however, the glycoside profiles were both quantitatively and qualitatively different from each other. Moreover, the authors demonstrated a relationship between metabolomic and phylogenetic data. Their obtained results show the possibility of effectively using MS-based metabolomic profiling for chemotaxonomy purposes in sea cucumbers.

The MALDI MS analysis of triterpene glycosides of *Holothuria scabra* revealed six major compounds saved during the processing of the body wall [97]. Mitu et al. showed that *H. scabra* releases triterpene glycosides into the surrounding seawater [100]. The characterization of these compounds by LC-multimode source MS led to the annotation of sixteen new and known compounds. A recent study used MALDI MS and LC-IM MS to characterize the triterpene glycoside composition of the viscera of *H. scabra* [107]. A combined analysis revealed 26 sulfated triterpene glycosides.

MALDI MS was used for the rapid structural characterization of triterpene glycosides in the body wall and Cuvierian tubules of the sea cucumber *Holothuria sanctori* [98]. Mass spectrometry analysis revealed eighteen triterpene glycosides, including eight novel compounds. Body wall triterpene glycosides showed higher diversity than those from the Cuvierian tubules.

LC-MS profiling of the sea cucumber *Eupentacta fraudatrix* revealed 26 sulfated, 18 non-sulfated, and 10 disulfated triterpene glycosides [99]. Many novel compounds were characterized by tandem MS, including those with previously unknown oligosaccharide chain types. Two new glycosides were isolated and their tentative structures were confirmed by NMR. Based on the literature data and obtained results, a biosynthetic pathway for oligosaccharide fragments of *E. fraudatrix* glycosides was proposed. LC-MS analysis of extracts from the respiratory trees, body walls, gonad tubules, guts, and aquapharyngeal bulbs indicated triterpene glycosides in all body components, although quantitative variability for certain triterpene glycosides was observed.

A combined technique involving microscopic analysis, MALDI MS, and MALDI MSI was used to study triterpene glycoside localization in the body wall of *Holothuria leucospilota* [102]. MALDI MS analysis of the body wall and the epidermal tissue extracts revealed twelve triterpene glycosides. The following MALDI MSI analysis showed the presence of detected glycosides in the epidermis of *H. leucospilota*, whereas in the dermis the circular and longitudinal muscle bands had no glycosides.

The composition of triterpene glycoside in the sea cucumber *Holothuria atra*, collected in the Persian Gulf, was studied using a modern analytical approach combining LC-MS, molecular networking, pure compound isolation, and NMR spectroscopy. In order to evaluate the entire pool of triterpene glycosides, the purified extract was subjected to LC-MS analysis using a column with pentafluorophenyl phase. The obtained MS data were used to create a molecular network using the GNPS Molecular Networking website. As a result, twelve triterpene glycosides were found, including three novel glycosides. Four major triterpene glycosides were isolated by HPLC, and their structures were confirmed by NMR [103].

The metabolic profiling of the polar fractions obtained from MTBE-based LLE of six sea cucumbers allowed for the identification of two sulfated glycosides found in all species and two species-specific nonsulfated glycosides [105]. Metabolic profiling was used for the screening of crude extracts of nine tropical sea cucumber species related to anti-fouling activities [106]. LC-MS analysis detected glycosides in all extracts; in total 102 triterpene glycosides were detected. The extract of *Bohadschia argus* represents one of the most active anti-fouling extracts, and includes 23 glycosides. The obtained results demonstrate that anti-fouling activities in sea cucumber extracts are species-specific and related to both triterpene glycoside total concentration and structures of presented triterpene glycosides. In another study, the relation between anti-fouling activities and the presence of triterpene glycosides was observed [125]. The UPLC-MS approach was used to evaluate the glycoside composition of *Apostichopus japonicus* [104]. As a result, five triterpene glycosides were detected and the variability of content of identified triterpene glycosides among the different types of *A. japonicus* was described.

The results of the aforementioned metabolomics studies indicate a huge structural variability of triterpene glycosides, as in the case of starfish polar steroids. Interestingly, certain species contain only a few triterpene glycosides, while dozens of glycosides have been found in the extracts of others. The maximum number of triterpene glycosides was detected in the extract of *E. fraudatrix*, with 54 compounds [99]. It should be noted that certain researchers used only MALDI MS methods, which do not distinguish between isomeric compounds in mixtures; thus, the real number of structures may be somewhat higher. Several studies have demonstrated the strict taxonomic specificity of the chemical composition of studied systematic groups of sea cucumbers [96,105]. Thus, MS-based metabolomic profiling can easily be used for chemotaxonomy purposes, both to clarify the species identity of unknown specimens and to confirm or revise the status of taxa.

The application of MALDI MSI and LC-MS profiling of extracts from various organs provides a better understanding of the distribution and localization of triterpene glycosides in animal tissues. Although glycosides are present in all organs, their distribution is heterogeneous [99]. Maximum total concentrations and number of structures can be found for the body walls and Cuvierian tubules [94,98,101]. By using MS-based methods, it has been found that sea cucumbers secrete toxic triterpene glycosides into the surrounding water [95]. These facts confirm the suggestion that triterpene glycosides have multiple defensive roles, including defense against predators and protection from parasites and microorganisms.

### 3.3. Starfish and Sea Cucumber Lipids

Marine invertebrates are known to be a valuable source of bioactive and dietary lipids, which are connected to the prevention of diseases and have applications in nutrition, cosmetics, pharmacy, and other fields. In this respect, an essential part of modern lipidomic studies is focused on fatty acids, glycerophospholipids, and sphingolipids in marine organisms such as sponges, cnidarians, worms, molluscs, and arthropods. Regarding starfish and sea cucumbers, research interest is largely linked to studying fatty acid composition and bioactive sphingolipids, including unique cerebrosides and gangliosides [126].

Sea cucumbers and starfish contain fatty acids in relatively small amounts [127]. Sea cucumber fatty acids usually account for less than 8% of their total weight, enriched in unsaturated fatty acids that may account for up to 70% [128]. The main fatty acids of sea cucumbers are 20:4 (n-6), 20:1, 20:5 (n-3), 16:0 and 18:0 [129]. Starfish are characterized by a high level of polyunsaturated acids, among which 20:5 (n-3) and 20:4 (n-6) are dominant [130]. MS-based analysis of fatty acids can be considered a well-established approach in view of both sample preparation and analysis protocols. Nowadays, GC-MS with chemical derivatization is routinely used to obtain an exhaustive view of the composition and metabolism of fatty acids. Analytical approaches and scientific results on fatty acid composition in marine invertebrates, including sea cucumbers and starfish, are discussed in detail in [13,131,132,133]. Fatty acid and sterol compositions of numerous sea cucumber [134] and starfish species [135] have been analyzed using GS-MS-based approaches. In addition, the variability in fatty acid content studied by GC-MS is used to analyze the effects of diet [136] and geographical origin [137,138] and to distinguish between wild and cultured animals [139].

The major phospholipids in Echinodermata include phosphatidylcholine, phosphatidylethanolamine, and phosphatidylserine. Sea cucumbers are characterized by high phosphatidylinositol content, while their lysophosphatidylcholine, lysophosphatidylethanolamine, diphosphatidylglycerol, phosphatidic acid, and phosphatidylinositol-4-phosphate contents are low [140].

Sample preparation for phospholipid determination in sea cucumbers and starfish is commonly based on extractions from the fresh whole body, body walls, or viscera via the classic Folch method [141], the Bligh and Dyer method [142], or extraction using a solvent combination of MTBE/MeOH/H_2_O [105] (Table 2 and Table S2). Different phospholipid headgroups and alterations in chain length, amount and position of double bonds in fatty acids, and ester bond types lead to an extremely complex composition of phospholipid mixtures. Traditionally, protocols that use deacylation and derivatization followed by GC or GC-MS identification were used to establish the fatty acid composition of phospholipids. However, such approaches are associated with the destruction of the original structures. In contrast, LC-MS methods allow for the detailed investigation of complex lipid mixtures in a high-throughput manner without prior purification and chemical modification. However, good chromatographic separation is critical for the accurate identification of lipids in such complex mixtures. Most frequently used reverse-phase sorbents, such as C8 or C18, allow for the separation of the molecules of phospholipids according to chain length and degree of saturation of acyl fatty acids. The use of HILIC or normal-phase (NP) columns, on the other hand, allows for the separation of molecules based on the structure of the polar head groups.

**Table 2:** Selected examples illustrating MS-based approaches for the analysis of starfish and sea cucumber lipids *.

Species Name	Extraction	Purification Method	MS Approach	Research Findings	Number of Analytes	Ref.
*Acaudina molpadioides*, *Cucumaria frondosa*, *Apostichopus japonicus*	CHCl_3_/MeOH	LLE, CC	RPLC-ESI-ITTOF MS	Cerebroside compositions of three sea cucumber species were characterized; many novel glucocerebroside structures were described	Cerebroside molecular species: *A. japonicus*—26, *C. frondosa*—40, *A. molpadioides*—12	[143]
*A. japonicus*, *Thelenota ananas*, *A. molpadioides*, *Bohadschia marmorata*	CHCl_3_/MeOH	LLE, SPE	RPLC-ESI-QTOF MS	A series of cerebrosides from four sea cucumber species were detected and annotated; the relation of long-chain base structures and fatty acids to sea cucumber genera were described	Cerebroside molecular species: *A. japonicus*—55, *T. ananas*— 107, *A. molpadioides*—87, *B. marmorata*— 75	[59]
*Pearsonothria graeffei*	CHCl_3_/MeOH	LLE, SPE	RPLC-ESI-QTOF MS	A series of cerebrosides of the sea cucumber *P. graeffei* were detected and annotated; characteristic structural features of sea cucumber cerebrosides were described	89 cerebroside molecular species	[144]
*C. frondosa*	CHCl_3_/MeOH/H_2_O	LLE, CC	RPLC-HESI-QOrbitrap MS	The sphingolipid composition of the sea cucumber *C. frondosa* was investigated; the relationship between sea cucumber sphingolipid structures and pro-apoptotic activities was discussed	35 cerebroside molecular species, 8 ceramide molecular species, 2 sphingosines	[145]
*Asterias amurensis*	Bligh and Dyer protocol	CC	RPLC-ESI-ITTOF MS	Cerebroside composition and distribution in viscera of the starfish *A. amurensis* were investigated; the potential usefulness of starfish as a source of raw material for cerebrosides was discussed	23 cerebrosides molecular species	[146]
*Parastichopus califormicus*, *C. frondosa*, *Isostichopus fuscus*, *Holothuria mexicana*, *H. polli*, *Bohadschia marmorata*	Bligh and Dyer protocol		NPLC-ESI-TripleTOF MS	A series of phospholipids, including rare representatives, were detected and annotated; qualitative and quantitative variations between sea cucumber species were established; the possibility of using phospholipid data for classification was shown	From 295 to 445 molecular species from 7 phospholipid classes (PG, PE, PI, PS, LPE, PC, LPC)	[142]
*B. marmorata*, *I. fuscus*, *H. polli*, *H. mexicana*, *C. frondosa P. califormicus*	H_2_O	LLE, SPE	HILIC LC-HESI-QOrbitrap MS	Seventeen ganglioside subclasses, including rare and new ganglioside structures, were discovered in six sea cucumber species; variations and characteristic features of the ganglioside composition of sea cucumbers were described	17 ganglioside subclasses	[147]

* Abbreviations: CC, column chromatography; ESI, electrospray ionization; HESI, heated electrospray ionization; HILIC, hydrophilic interaction chromatography; IT, ion trap; LC, liquid chromatography; LLE, liquid-liquid extraction; LPC, lysophosphatidylcholine; LPE, lysophosphatidylethanolamine; NPLC, normal-phase liquid chromatography; MS, mass spectrometry; PC, phosphatidylcholine; PE, phosphatidylethanolamine; PG, phosphatidylglycerol; PI, phosphatidylinositol; PS, phosphatidylserine; QOrbitrap, quadrupole-Orbitrap; QTOF, quadrupole time-of-flight; RPLC, reverse-phase liquid chromatography; SPE, solid-phase extraction; TOF, time-of-flight.

Although phospholipids have drawn intense interest in recent years as substances with the potential for human health benefits, and although modern LC-MS lipidomic methods have been used extensively, only limited studies are available on the phospholipid content of starfish and sea cucumbers [148]. The investigation of the composition of phospholipids in several echinoderm species by LC-ESI MS revealed the predomination of alkylacyl-PC and alkenylacyl-PE forms in starfish and sea cucumbers [141]. Structural characterization was achieved by comparing the retention times and in-source fragmentation patterns obtained in negative and positive ion modes with single-stage MS. The NPLC-ESI TripleTOF MS was employed to study the phospholipid composition of the dried body walls of six sea cucumber species [142]. The application of normal-phase liquid chromatography as a separation technique allowed for the division of complex mixtures into subclasses with reproducible retention times as well as the separation of isobaric molecules in the same subclass. As a result, between 295 to 445 molecular species belonging to eleven phospholipid subclasses (phosphatidylcholines, phosphatidylserines, phosphatidylethanolamines, phosphatidylinositol, phosphatidic acids, phosphatidylglycerols, lysophosphatidylcholines, lysophosphatidylethanolamine, lysophosphatidylserine, lysophosphatidylinositol, and rare phosphonoethanolamine) were detected in each species. Identification and structure elucidation were based on retention times, ion forms, and specific fragmentation patterns obtained in negative ion mode using the LIPIDMAPS database. Semiquantitation followed by statistical analysis demonstrated differences in the phospholipid profiles of the studied species. Analysis of the nonpolar fraction obtained by extraction of six Egyptian sea cucumber species was performed using UPLC-Orbitrap MS in positive and negative ion modes [105]. For metabolite identification, the obtained data (*m*/*z*, retention time, isotope and fragmentation patterns) were searched against public databases. A total of fifteen free fatty acids and 45 triacyl glycerols were detected. Statistical analysis showed that quantitative variations in lipid content between the studied species were associated with habitat or food changes, not with taxonomical relationships.

Sphingolipids constitute an extremely diverse class of bioactive polar lipids. As components of cell membranes and intracellular mediators, sphingolipids are involved in cell recognition and signal transduction processes [149,150]. Sphingolipids from marine organisms exhibit various activities, including antitumor, immunomodulatory, antiviral, neuritogenic, and other activities [126,151].

**Figure 6:**
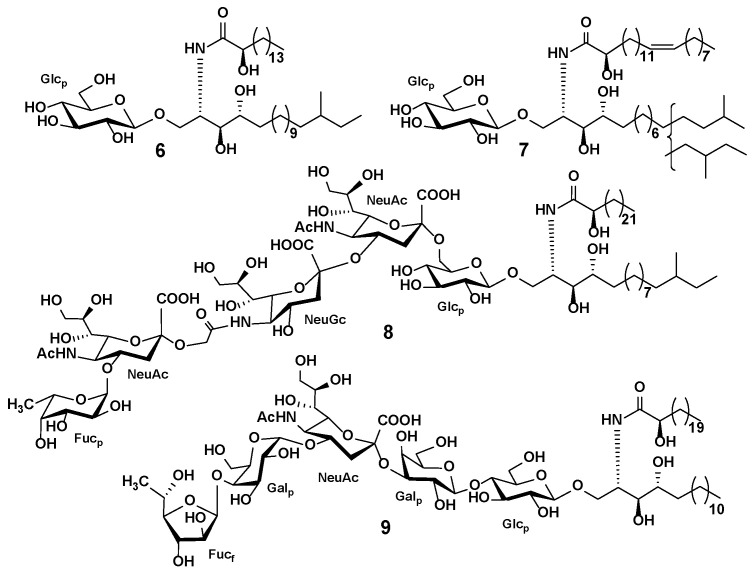
The structures of typical echinoderm cerebrosides (luidiacerebroside A (**6**) from the starfish *Luidia maculata* [155] and glucocerebroside HPC-3-A (**7**) from the sea cucumber *Holothuria pervicax* [156]) and gangliosides (ganglioside molecular species from the sea cucumber *H. pervicax* (**8**) [157] and acanthaganglioside I (**9**) from the starfish *Acanthaster planci* [158]).

Sphingolipids, particularly cerebrosides and gangliosides, are important components of echinoderm lipids that have drawn attention because of their structure and bioactivity. Compared to those present in mammals and plants, the sphingolipids present in echinoderms have notable structural differences. Variations in chain length and in the degree of saturation and/or hydroxylation of the sphingoid backbone and fatty acids lead to the extensive variety of cerebrosides structures. Starfish and sea cucumber gangliosides remain little studied. These compounds have a specific sugar core, including sialic acids within carbohydrate chains, as well as additional monosaccharide residues and unusual types of glycosidic bonds between them (Figure 6) [126]. The determination of their diverse structures and variety of sphingoid backbones are very important for understanding the functional and nutritional significance of dietary sphingolipids. To date, about 150 sphingolipids from fifteen starfishes and nine sea cucumbers have been studied, several of which have demonstrated biological activity [126]. Cerebrosides from sea cucumbers and starfish show activity against nonalcoholic fatty liver disease [152] and an inhibitory effect on cell proliferation through the induction of apoptosis in cancer cells [153]. Sea cucumber sphingosine has strong cytotoxicity against colon cancer cells [154]. Gangliosides of starfish and sea cucumbers show slight neuritogenic activity. However, the bioactivity of these compounds remains poorly investigated [126].

Regarding cerebroside extraction from starfish and sea cucumbers, LLE with methanol:chloroform mixtures is preferred, although extraction following the Bligh and Dyer method or modifications of it is applicable. Subsequent purification of crude extracts by LLE, SPE, or silica gel column chromatography provides pure fractions containing cerebrosides. High-speed counter-current chromatography (HSCCC) has recently been pointed out as a way to improve the resulting cerebroside fractions without requiring extraction procedures or additional purification [145]. Gangliosides, as the more polar compounds, can be extracted from sea cucumber body walls by homogenization in water followed by purification using LLE with a solvent combination of CHCl_3_/MeOH/ H_2_O followed by SPE purification using C8 sorbent [147] (Table 2 and Table S2).

Because of their structural complexity, sphingolipids are difficult to analyze using one method. Formerly, sphingolipids were quantified by thin-layer chromatography (TLC); structure elucidation comprised many stages of chemical decomposition and derivatization followed by GC, GC-MS, HPLC, MS, and NMR analyses [159]. Today, LC-MS with electrospray ionization is the main tool for the detection and annotation of sphingolipids, including both known and novel molecular species. RPLC with isocratic elution with 95% MeOH with 5 mM ammonium acetate and 0.05% acetic acid or elution with 95% ACN is commonly used to separate sphingolipids. HILIC-LC on the GOLD-amino column has been used in the separation of gangliosides from sea cucumbers [147]. In this approach, the ganglioside subclasses are eluted in a specific time range based on their amounts of sialic acid residues.

The ESI in positive ion mode is the most extensively used ionization technique for the analysis of cerebrosides (Table 2). Although the locations of the double bonds in the fatty acyl chain often cannot be unequivocally identified by CID, the specific fragment ions from fatty acid, sphingosine, and sugar units allow putative structures to be proposed [59,143,144,145]. Gangliosides detected in the negative ion mode mainly form deprotonated or double deprotonated ions. Characteristic fragment ions and neutral losses formed by oligosaccharide chain fragmentation reveal a monosaccharide composition and different sialic acid types [147].

The investigation of cerebrosides from various sources by LC-MS showed that the cerebroside composition of sea cucumbers differs from those of plants (maize, rice) and mushrooms (maitake). [160]. Research into the cerebroside composition of the sea cucumbers *Apostichopus japonicus*, *Thelenota ananas*, *Acaudina molpadioides*, *Bohadschia marmorata*, *Cucumaria frondosa*, and *Pearsonothria graeffei* using LC-MS led to the discovery of a large series of compounds and showed that sea cucumber sphingolipids are much more diverse than was conventionally thought [59,143,144,145] (Table 2). Each studied species contained several dozen cerebroside molecular species, with the most complicated composition belonging to *T. ananas*. Several sea cucumber species were found to have similar sphingolipid compositions, while the profiles of others were dramatically distinctive. The analysis of many structures allowed identification of the characteristic structural features of sea cucumber cerebrosides. A sphingoid base (d17:1) is typically predominant in sea cucumber cerebrosides and is not widely found in plants, mammals, or fungi. In addition, the occurrence of C23:1h is characteristic of sea cucumber cerebrosides and is rarely found in plants, mammals, or fungi. The FA contained in cerebrosides from sea cucumbers is similar to those of common mammals, although it has more double bonds and hydroxylation. The study of the cerebroside composition of the gonads, viscera, and whole body of the starfish *Asterias amurensis* revealed a characteristic structure distribution that can be divided into three major structural groups [146].

HILIC-ESI MS was used to identify gangliosides in six sea cucumber species. Seventeen ganglioside subclasses were detected, and their oligosaccharide chains were characterized by tandem MS [147]. The results indicated that sea cucumber gangliosides differ from mammalian gangliosides in monosaccharide composition, number, and types of sialic acids. Moreover, gangliosides with phosphoinositidyled sialic acid and tetrasialogangliosides were identified in sea cucumbers for the first time.

### 3.4. Multi-Class Profiling Studies

In contrast to the single-class approach, the multi-class approach attempts to simultaneously detect the qualitative and quantitative characterization of various metabolites of different chemical groups in a single analysis run. Essentially, research that uses a multi-class approach aims to investigate crude extracts or study the composition of bioactive fractions. Due to the complicated composition of marine invertebrates, such extracts or fractions contain a wide diversity of compounds. The chemical pool of such extracts depends primarily on the solvent used and/or on the extraction protocol. Methanol, ethanol, dichloromethane, chloroform, ethyl acetate, and their mixtures are often used for extraction. Non-polar solvents extract complex mixtures of fatty acids, lipids, carotenoids, triterpenes, and sterols, while the use of polar solvents leads to extracts containing mixtures of polar steroid compounds or triterpene glycosides, polar lipids, and other compounds. The results depend on the analytical method and platform. The widely used multi-class GC-MS approach is used for analysis and accurate identification via a database search of sterols, triterpenes, and fatty acids such as methyl esters. LC-MS is used to characterize more polar compounds.

The LC-MS metabolic profiling of sea cucumber *Holothuria spinifera* extract indicated secondary metabolites of several classes [161]. Gradient elution on the C18 column with ESI-Orbitrap MS operating in positive and negative ion modes was used for the investigation of methanol:dichloromethane extract. It is worth noting that only 4% of metabolites were detected in either mode. The molecular formula was predicted using the MZmine algorithm and identification was achieved using MarinLit and the Dictionary of Natural Products databases. As a result, thirteen secondary metabolites belonging to the fatty acids, phenolic diterpenes, and triterpenes were identified.

Investigation of antifouling and antibacterial activities of three extracts from different organs of the sea cucumber *Holothuria leucospilota* showed that ethyl acetate extract of the body wall possessed the most pronounced activity [162]. In order to determine its bioactive compounds, GC-MS was used. Using the NIST GC-MS library, seventeen metabolites, including five terpenes and terpenoids and six fatty acids, were identified.

The lipid, fatty acid, and sterol compositions of four sea cucumbers were analyzed to assess their feeding habits [163]. The extracts were obtained using a modified Bligh and Dyer protocol and analyzed using a thin-layer chromatography-flame ionization detector (TLC-FID) analyzer to quantify lipid classes and GC-MS for the identification of individual metabolites. The sea cucumbers were found to be rich in phytosterols and algal-derived fatty acids, suggesting tight trophic coupling to phytodetritus, while the relatively large proportions of stanols were probably the result of enteric bacteria. GC-MS was used for targeted profiling of fatty acids as methyl esters and amino acids after sample derivatization with N-methyl-N-tert-butyldimethylsilyltrifluoroacetamide (BSTFA) and trimethylchlorosilane (TMCS) of three sea cucumber species [127].

Dichloromethane, methanol, and aqueous extracts of *Linckia laevigata*, *Fromia indica*, *Cryptasterina pentagona*, and *Archaster typicus* were tested in order to identify surface-bound metabolites that protect the starfish from fouling; the most biologically active fractions were analyzed by GC-MS [164]. Several fatty acids and sterols were identified using the NIST and Wiley GC-MS databases. GC-MS analyses of the surface-extracted metabolites of each starfish specimen identified hexadecanoic acid, cholesterol, lathosterol, and sitosterol as the compounds responsible for the antifouling effects.

Pereira et al. proposed the GC-MS method for simultaneous analysis of fatty acids, amino acids, sterols, and lupanes in marine animals and applied this method to the characterization of the starfish *Marthasterias glacialis* extract [165]. Using ethanol as the extraction solvent at 40 °C and N-methyl-N-(trimethylsilyl)-trifluoroacetamide (MSTFA) as the derivatization reagent, forty compounds (including fifteen amino acids, sixteen fatty acids, six sterols, and three lupanes were detected and quantified.

The LC-MS approach was used for the profiling and identification of the active compounds of dichloromethane:methanol extract of the holothurian *Pseudocolochirus violaceus*, which exhibited strong antiproliferative effects [166]. The mixture was separated on a C18 column and metabolites were detected using ESI MS in positive and negative ionization modes. Compound identification was performed by accurate mass measurement and comparison of the obtained data with previously reported information. As a result, 24 compounds, belonging mainly to the terpenes, steroids, and fatty acids, were detected using both positive and negative ionization modes, several of which have previously been reported to exhibit antiproliferative capacity in cancer cells.

A combination of the GC-MS and LC-MS methods was applied to obtain a comprehensive view of the composition of ethyl acetate extract of the sea cucumber *Holothuria forskali* [167]. The GC-MS analysis revealed 25 major components identified by the NIST GC-MS library, while LC-MS showed eight molecules, including fat-soluble vitamins, phytosterols, and phenolic acids, identified by comparison of retention times and MS data obtained for pure standards.

Zakharenko et al. proposed an alternative method for the extraction of bioactive compounds from sea cucumbers using a two-step process with carbon dioxide extraction followed by extraction with CO_2_ and ethanol as a co-solvent [168]. The obtained extracts were tested by LC-IT MS in negative and positive ion modes and tandem MS. The identification of the metabolites was achieved using the Bruker library database and literature data, and revealed the presence of fifteen triterpene glycosides, eighteen styrene compounds, and fourteen carotenoids.

Variability in the chemical composition of the viscera and body walls of the sea cucumber *A. japonicus* extracted by methanol was investigated by the LC-MS approach, and 85 metabolites were determined [169]. Multivariate data analysis using PCA and PLS-DA revealed significant differences between the viscera and body walls. To identify the main characteristic compounds of viscera, several sphingoid-based nucleoside analogs were isolated and their structures were confirmed by MS/MS and NMR methods.

The investigation of sea cucumber metabolites is not restricted to metabolite profiling of extract, and can be applied to the profiling of volatile compounds as well. Volatile compounds of eight dried sea cucumber species with different geographical origins were analyzed using a combination of headspace solid-phase microextraction and GC-MS [170]. Metabolite identification was achieved by matching data with NIST and Wiley GC-MS databases and confirmed by comparing retention times and mass spectra with standard compounds. As a result, 42 volatile compounds, including aldehydes, alcohols, aromatic compounds, and furans, were identified in the dried sea cucumbers, several of which were determined as odour-active compounds.

## 4. Applications of Metabolome-Oriented Approaches in Studies of Starfish and Sea Cucumbers

MS-based targeted metabolomics were applied to investigate the influence of different environmental factors on the polar steroids of the starfish *P. pectinifera* [87]. Extracts of control starfishes and starfishes exposed to water heating, oxygen deficiency, feeding, injury, and different water salinity levels were purified by SPE and analyzed using LC-ESI-QTOF MS. An in-house library of retention times and MS data on previously characterized polar steroid metabolites of *P. pectinifera* were used for metabolite identification. Univariate and multivariate statistical analyses revealed variations in the steroid metabolome between the control and treatment groups. In order to further evaluate stress-induced differences, PCA and PLS-DA analyses were carried out on each group of starfish individually with the control group. The results revealed that differences caused by feeding, injury, and heating were greater than in the other starfish groups. These states had similarities in their effects on the steroid metabolome of starfish. Most asterosaponins were reduced, and most polyhydroxysteroids and related glycosides were increased. These differences in steroid metabolite profiles may relate to the biological multifunctionality of these compounds.

MALDI MSI was applied to study the precise localization of sea cucumber *H. forskali* triterpene glycosides in the Cuvierian tubules of control and stressed sea cucumbers [93]. Stressed animals were mechanically disturbed for 4 h by repetitive hitting using a specific device. Statistical multivariate tests using PCA showed statistical differences in triterpene glycoside composition between the control and stressed groups. Triterpene glycosides with corresponding ions at *m*/*z* 1287 and 1303 were mainly localized in the connective tissue of the tubules of both control and stressed sea cucumbers. Glycoside ions at *m*/*z* 1125 and 1141 were present in relaxed animals, while ions at *m*/*z* 1433, 1449, 1463, and 1479 were observed in the Cuvierian tubules of stressed animals in the outer part of the connective tissue. The authors proposed that the latest glycosides are stress-specific compounds formed by modifications of the glycosides with shortened oligosaccharide chains. Another study revealed that *H. forskali* releases glycosides into the surrounding seawater. Among these secreted glycosides, holothurinoside G was detected in the seawater surrounding relaxed sea cucumbers, while holothurinosides C, F, M, L, and desholothurin A were secreted when the animals were stressed [95].

The most extensive metabolomics research has been performed on the commercially important sea cucumber *Apostichopus japonicus* (Table S3). Most of these studies used a non-targeted UPLC-QTOF MS approach and focused on primary metabolites such as amino acids, sugars, fatty acids, and common metabolites, and did not involve sea cucumber-specific triterpene glycosides and gangliosides.

High temperature and low oxygen concentration are the common environmental stress factors for marine invertebrates, and their impact on *A. japonicus* has been studied using MS-based metabolomics [171]. Changes in the concentrations of 84, 68, and 417 metabolites related to the responses to heat, hypoxia, and combined stress, respectively, were detected by LC-MS and multivariate statistical analysis. Among the detected metabolites, compounds atypical for echinoderms such as the plant glycoside tokoronin, the synthetic drug tirofiban, and others were found, which may be due to identification errors (the authors did not provide information on how metabolite identification was performed). Another investigation of acute hypoxia in *A. japonicus* showed that levels of most lipids increased with the elongation of hypoxia. These results imply that the homeostasis of synthesis and degradation of lipids and their derivatives are strongly affected by hypoxic stress [172]. Liu et al., used GC-MS to compare the metabolic profiles of a thermotolerant strain of *A. japonicus* with a control group, and found significant differences in the concentrations of 52 metabolites [173]. Evisceration is a well-known stress response of sea cucumbers, although the biochemistry of this process is unclear. Metabolomic analysis of coelomic fluids ejected during *A. japonicus* evisceration using LC-MS followed by univariate and multivariate analysis revealed five significantly changed signaling pathways [47]. In response to high temperatures, sea cucumbers can enter a state characterized by inactivity, cessation of feeding, gut degeneration, and decreased metabolic rate. This physiological state is called aestivation. Yang et al. used transcriptomic and metabolomic approaches to explore alterations in *A. japonicus* during the aestivation stage [174]. LC-MS analysis revealed that downregulated metabolites were associated with fatty acid metabolism, carbohydrate metabolism, and the TCA cycle. UPLC-QTOF MS was used to describe the metabolic changes induced by skin ulceration syndrome, the main disease affecting the development of *Apostichopus japonicus* in the aquaculture industry [175]. As a result, variations in metabolites mainly related to amino acid metabolism, energy metabolism, immunity, osmoregulation, and neuroactive ligand-receptor interaction has been discovered.

Another research focus has been directed towards the study of metabolomic changes induced by the impact of various factors. LC-MS has been used to highlight metabolomic differences between cage-cultured, pond-cultured, and bottom-sowed *A. japonicus* [176]. Multivariate analysis and enrichment of metabolic pathway analyses revealed differential metabolites participating in lipid, amino acid, carbohydrate, and nucleotide metabolism. The investigation of *A. japonicus* coelomic fluids in different sexes and reproductive states by UPLC-QTOF MS and multivariate statistical analysis revealed variations in phenylalanine metabolism and unsaturated fatty acid synthesis [177]. LC-MS highlighted significant metabolic differences in the muscle tissue of animals between the nonbreeding and growth stages [178]. The metabolite profiles obtained using UPLC-QTOF-MS of four *A. japonicus* varieties (green, white, purple, and spiny) were compared, and differences were identified using multivariate analysis [179]. Differential metabolites included fatty acids, amino acids, phospholipids, and sugars. In another study, a similar approach was applied to reveal the metabolic changes in white, green, and purple *A. japonicus* body walls during the pigmentation process [180]. Statistical analysis differentiated the body wall chemical composition among the three color morphs, and thirteen annotated metabolites showed significant differences in white, green, and purple sea cucumbers. UPLC-QTOF MS metabolomic profiling was applied to distinguish *A. japonicus* from different geographical origins [181]. Data analysis using OPLS-DA showed that differential metabolites mainly included amino acids and lipids.

Melatonin-induced metabolomic changes in the muscle tissues of *A. japonicus* have been tested using UPLC-QTOF MS [182]. Statistical analysis with PCA, PLS-DA, fold-change analysis, and *t*-test showed alterations in the levels of 22 different metabolites, including serotonin, retinoic acids, and fatty acids, which can explain the observed sedative effect of melatonin on this species. The LC-MS metabolomic analysis revealed that pedal peptide-type neuropeptides involved in the regulation of locomotor behavior in *A. japonicus* induce changes in the levels of certain phospholipids [183].

Most of the mentioned metabolomics studies of *A. japonicus* used a similar workflow involving the extraction of tissue samples with methanol, methanol:water, or methanol:acetonitrile mixtures, homogenization, centrifugation, and LC-MS analysis mainly using RP separation and ESI-QTOF mass spectrometers for the detection of metabolites operating in the mass ranges from 50 to 1200 Da in positive and negative ion modes. Although a huge number of metabolites (from several dozen to 4435 metabolites) are found in almost every work, the compounds that are mainly responsible for the bioactive properties of sea cucumbers (triterpene glycosides, cerebrosides, and gangliosides) remain undetected, and their variations under the studied conditions remain unclear. At the same time, the results of the other works [87,93] indicate statistically significant changes in the levels of specific metabolites, namely, starfish polar steroids and sea cucumber triterpene glycosides, in response to stresses and environmental factors. Thus, the influence of many factors and physiological phenomena, such as aestivation and evisceration, on large groups of bioactive metabolites remains unexplored. It is well known that starfish and sea cucumbers have extraordinary regenerative potential, however, the features of this process remain poorly explored at the metabolome level.

## 5. Conclusions and Perspectives

The unique features of biosynthesis and metabolism and the diversity of their metabolites explain the researchers’ interest in these organisms. Starting in the middle of the last century, chemical research on echinoderm metabolites has resulted in hundreds of compounds, many of which have demonstrated biological and pharmacological effects. The structural elucidation of the bioactive compounds of starfish and sea cucumbers is a difficult task, combining isolation of pure compounds with modern MS and NMR techniques to unambiguously determine the structures of new compounds.

MS-based metabolomics approaches have proven to be a powerful research tool in the natural product area. The application of MS-based techniques has made it possible to study chemical compounds without the laborious process of isolating individual compounds. Using modern metabolomics methods in the marine sciences allows evaluation of the biochemical diversity of marine systems and expands our understanding of the chemical space of marine compounds.

To date, only a few dozen species of starfish and sea cucumbers have been studied using MS-based metabolomics approaches, a very small fraction of the more than 3600 known species. Most of the studied species are aquaculture species or readily available and widely distributed species, and most deep-sea and rare species remain almost unexplored.

In summarizing MS-based metabolomics studies on the bioactive secondary metabolites of starfish and sea cucumbers, it was found that this approach allows for the ready detection and annotation of polar steroids, triterpene glycosides, and lipids in producer organisms. Obtained data allow for their exact or preliminary structures to be proposed. The data obtained thus far make it possible to assess the prospects for the search for new bioactive molecules as well as to draw conclusions about their taxonomic distribution, biogenesis, and biological functions. These methods are used to compare metabolomic profiles of different echinoderm species and populations in ecological, dietary, and biosynthesis studies.

The main difficulties of applying MS-based metabolomics approaches are related to the extreme complexity of the mixtures being analyzed. However, the use of modern chromatography and mass spectrometry methods allows these methods to be successfully applied. UPLC with analytical columns packed with sub-2-µm sorbents allows metabolites with closely related structures to be separated. The introduction of cutting-edge mass-spectrometry techniques such as ion mobility, improved ion dissociation techniques, and ultra-high resolution mass analyzers can provide high-performance MS analyses. While identification of detected peaks remain the main problem in MS data analysis, constructing specialized databases of echinoderm metabolites and improving in silico computational algorithms may improve the obtained results considerably.

## Supplementary Materials

The following supporting information can be downloaded at: https://www.mdpi.com/article/10.3390/md20050320/s1, Table S1: Instrumental and methodological details of MS-based applications for the analysis of starfish polar steroids and sea cucumber triterpene glycosides; Table S2: Instrumental and methodological details of MS-based applications for the analysis of starfish and sea cucumbers lipids; Table S3: Instrumental and methodological details in metabolomics studies of starfish and sea cucumbers.
