# Supplementary material for: Application of MS-Based Metabolomic Approaches in Analysis of Starfish and Sea Cucumber Bioactive Compounds
Source: Mar Drugs. 2022 May 12;20(5):320. doi: 10.3390/md20050320 (PMC9147407; doi:10.3390/md20050320)
Supplement: Supplementary file 1 [file marinedrugs-20-00320-s001.zip › marinedrugs-1710959-supplementary.pdf]

Supplementary Materials:

Review

Application of MS-based metabolomic approaches in analysis of starfish and sea cucumber bioactive compounds

Roman S. Popov*, Natalia V. Ivanchina, Pavel S. Dmitrenok*

[1] G.B. Elyakov Pacific Institute of Bioorganic Chemistry, Far Eastern Branch of Russian Academy of Sciences, 159 Prospect 100-let Vladivostoku, Vladivostok 690022, Russia; ivanchina@piboc.dvo.ru (N.V.I.)

* Correspondence: popov_rs@piboc.dvo.ru (R.S.P.); paveldmt@piboc.dvo.ru (P.S.D.); Tel.: +7-423-231-1132 (P.S.D.)

Contents

Table S1. Instrumental and methodological details of MS-based applications for the analysis of starfish polar steroids and sea cucumber triterpene glycosides.

Table S2. Instrumental and methodological details of MS-based applications for the analysis of starfish and sea cucumber lipids.

Table S3. Instrumental and methodological details in metabolomics studies of starfish and sea cucumbers.

Abbreviations

ANOVA, Analysis of variance; **APPI**, Atmospheric pressure photoionization; **BSTFA**, N-methyl-N-tert-butyldimethylsilyltrifluoroacetamide; **CC**, Column chromatography; **CID**, Collision-induced dissociation; **CSI**, Captive spray ionization; **EI**, Electron ionization; **ESI**, Electrospray ionization; **FAB**, Fast atom bombardment; **FTICR**, Fourier-transform ion cyclotron resonance; **GC**, Gas chromatography; **HESI**, Heated electrospray ionization; **HILIC**, Hydrophilic interaction chromatography; **HPLC**, High-performance liquid chromatography; **HSCCC**, High-speed counter-current chromatography; **IM**, Ion mobility; **IT**, Ion trap; **LC**, Liquid chromatography; **LLE**, Liquid-liquid extraction; **LPC**, Lysophosphatidylcholine; **LPE**, Lysophosphatidylethanolamine; **NMR**, Nuclear magnetic resonance; **NPLC**, Normal-phase liquid chromatography; **MALDI**, Matrix-assisted laser desorption/ionization; **MS**, Mass spectrometry; **MS/MS**, Tandem mass spectrometry; **MSI**, Mass spectrometry imaging; **MSPD**, Matrix solid-phase dispersion; **MSTFA**, N-methyl-N-(trimethylsilyl)-trifluoroacetamide; **MTBE**, Methyl tert-butyl ether; **nanoESI**, Nanoelectrospray; **PC**, Phosphatidylcholine; **PCA**, Principal component analysis; **PE**, Phosphatidylethanolamine; **PG**, Phosphatidylglycerol; **PI**, Phosphatidylinositol; **PLS-DA**, Partial least squares discriminant analysis; **PS**, Phosphatidylserine; **QOrbitrap**, Quadrupole-Orbitrap; **QTOF**, Quadrupole time-of-flight; **QQQ**, Triple-quadrupole; **RPLC**, Reverse-phase liquid chromatography; **SPE**, Solid-phase extraction; **TLC-FID**, Thin-layer chromatography-flame ionization detector; **TMCS**, Trimethylchlorosilane; **TOF**, Time-of-flight; **TOF/TOF**, Tandem time-of-flight; **FT**, Fourier transform; **UPLC**, Ultra-performance liquid chromatography; **V_{cap}**, capillary voltage.

Table S1. Instrumental and methodological details of MS-based applications for the analysis of starfish polar steroids and sea cucumber triterpene glycosides.

Species	Research focus	Extraction	Purification and desalting	Analytical approach	Separation and MS detection conditions	Statistical methods	Ref.
				**Asteroidea**			
Asterias rubens	Applying of LC-NMR-MS analytical approach for screening of an asterosaponin subfraction and characterization of new compounds	MSPD: animals were cut into small pieces, mixed with H_2O and LiChroprep RP-18 phase, ground in a mortar, and transferred into a C18 column; elution with 50% ACN yielded the asterosaponin fraction		LC-MS	RPLC: YMC J'sphere ODS L80 column (4.6×250 mm); isocratic elution at 0.05 or 0.5 mL/min flow rate of 20 mM NH_4HCO_2 in D_2O/ACN (65.5:34.5, *v:v*)	-	[84]
					ESI-IT MS (neg): V_{cap} – 3.15 kV; end-plate voltage – 2.35 kV; nebulization with N_2 at 30 psi; dry gas flow – 11 L/min at 300°C		
A. rubens	Structural characterization, studying the inter-organ spatial distributions and inter-individual variations of asterosaponin composition	Four animals were dissected into body wall (oral and aboral), gonads, stomach, and pyloric caeca and extracted with 90% MeOH	LLE: partitioned successively against *n*-hexane, CH_2Cl_2, and $CHCl_3$; Column chromatography: Amberlite XAD-4 column (washing with H_2O and elution with MeOH); LLE: partitioned against *i*-BuOH	LC-MS	RPLC: Kinetex C18 column (2.1×100 mm, 2.6 μm); isocratic elution at 0.1 mL/min flow rate and 30 °C of 80% B; A: 0.1% FA in H_2O; B: MeOH; run time: 15 min	-	[85,86]
					ESI-QQQ MS (neg): V_{cap} – 3.1 kV; cone voltage – 20 V; source temperature – 100 °C; desolvation temperature – 300 °C		
				MALDI	MALDI-QTOF MS (neg): nitrogen laser (337 nm); matrix – 2,5-DHB in H_2O/ACN with the addition of *N,N*-dimethylaniline		
					MALDI-TOF/TOF MSI (neg): carboxymethyl cellulose was used as an embedding medium; Smartbeam II (Nd-YAG, 355 nm) laser; matrix – 2,5-DHB in H_2O/MeOH		
Aphelasterias japonica	Profiling and structural characterization of polar steroid compounds	Five fresh animals were chopped and extracted with EtOH	SPE: Strata-X (polymeric reversed-phase), 33 μm, 60 mg/3 mL	LC-MS	RPLC: Zorbax 300SB-C18 column (1.0×150 mm, 3.5 μm); gradient elution at 0.05 mL/min flow rate and 40 °C; A: 0.1% HAc in H_2O; B: 0.1% HAc in ACN; run time: 45 min	-	[77]
					ESI-QTOF MS (neg&pos): V_{cap} – ±3.5 kV; nebulization with N_2 at 2 bar; dry gas flow – 5 L/min at 325°C; fragmentor voltage – 250 V (neg) and 360 V (pos)		
Patiria pectinifera	Profiling and structural characterization of polar steroid compounds	Twenty fresh animals were chopped and extracted with EtOH	SPE: Strata-X (polymeric reversed-phase), 33 μm, 60 mg/3 mL	LC-MS	RPLC: Zorbax 300SB-C18 column (2.1×250 mm, 5 μm); gradient elution at 0.15 mL/min flow rate and 40 °C; A: 0.1% HAc in H_2O; B: 0.1% HAc in ACN; run time: 30 min	-	[78,87]

					ESI-QTOF MS (neg&pos): V_{cap} – ±3.5 kV; nebulization with N_2 at 2 bar; dry gas flow – 5 L/min at 325°C; fragmentor voltage – 250 V (neg) and 360 V (pos); APPI-QTOF MS (neg): V_{cap} – 1.5 kV; nebulization with N_2 at 2 bar; dry gas flow – 7 L/min at 350°C; vaporizer temperature – 350 °C; fragmentor voltage – 90 V		
Luidia senegalensis	Profiling and characterization of polar steroids	Animals were chopped and extracted with 70% EtOH	SPE: Chromabond C18 ec, 500 mg	LC-MS	RPLC: Kinetex Core-Shell C18 column (2.1×100 mm, 1.7 μm); gradient elution at 0.2 mL/min flow rate; A: 0.1% FA in H_2O; B: 0.1% FA in MeOH; run time: 10 min<br>ESI-IT MS (neg&pos): spray voltage – 5 kV; capillary voltage – 35 V	-	[88]
Lethasterias fusca	Profiling, structural characterization, and studying of distribution of polar steroid compounds	Five fresh animals were chopped and subjected to the triple extraction with EtOH; another five animals were dissected into body walls, gonads, stomach, coelomic fluid, and pyloric caeca and organs were extracted with EtOH	LLE: twice extraction with a solvent combination of $CHCl_3$/MeOH/ H_2O (8:4:3, *v:v:v*); SPE: BondElut C18, 100 mg/1 mL	LC-MS	nanoRPLC: Acclaim PepMap RSLC C18 column (75 μm×150 mm, 2 μm); gradient elution at 400 nL/min and 40 °C; A: 0.1% FA in H_2O; B: 0.1% FA in ACN; run time: 81 min<br>CSI-QTOF MS (neg&pos): V_{cap} – 1.3 kV; dry gas flow – 3 L/min at 150 °C	ANOVA	[79,87]
Echinaster sepositus	Structural characterization, studying of distribution and variation of asterosaponin composition	Animals were dissected into the stomach, pyloric caeca, gonads, aboral body wall, and oral body wall and extracted by microwave-assisted extraction with 60% MeOH	Similar to [2]	ESI MS	ESI-QOrbitrap MS (neg&pos): spray voltage – 3-4 kV; capillary temperature – 300 °C; sheath gas flow rate – 10–80; auxiliary gas flow – 0–10	-	[90]
Heliaster helianthus	Characterization of the chemical composition of fractions that induced escape reactions and mortality in the limpets	Ten animals were dissected into arms and central disks and homogenized in EtOH	LLE: partitioned successively against cyclohexane and *n*-BuOH; Column chromatography: Sephadex LH-60 column (elution with MeOH:H_2O (2:1) give fractions	ESI MS	ESI-QTOF MS (neg): V_{cap} – 3.3 kV; cone voltage – 30 V; desolvation temperature – 100 °C	-	[91]

			containing mixtures of polar steroids				
					**Holothuroidea**		
Holothuria forskali	Structural characterization of *H. forskali* triterpene glycosides and comparison of glycoside composition of body walls and Cuvierian tubules	Animals were dissected into the body wall and Cuvierian tubules; organs were homogenized and extracted twice with 70% EtOH	LLE: partitioned successively against *n*-hexane, CCl_4, and $CHCl_3$; Column chromatography: Amberlite XAD-4 column (washing with H_2O and elution with MeOH); LLE: partitioned against *i*-BuOH	LC-MS	RPLC: Symmetry C18 column (4.6×150 mm, 5 µm); gradient elution at 1 mL/min flow rate and 27 °C; A: H_2O; B: MeOH; run time: 15 min	-	[92]
					ESI-QTOF MS (pos): V_{cap} – 3.1 kV; cone voltage – 50 V; nebulization with N_2; desolvation temperature – 300 °C; source temperature – 120 °C		
				MALDI MS	MALDI-QTOF MS (pos): nitrogen laser (337 nm); matrix – HCCA in acetone		
H. forskali	Investigation of triterpene glycoside localization in the Cuvierian tubules and comparison data from stressed and relaxed animals	Relaxed animals were anesthetized in a 0.1% solution of 1-phenoxy-2-propanol in seawater. Stressed individuals were mechanically disturbed by repetitive hitting. Cuvierian tubules from relaxed and stressed animals were collected and prepared for MALDI MS and MALDI MSI	Samples for MALDI MS were prepared similarly to [2]	MALDI MS	MALDI-TOF/TOF MS (pos): Smartbeam (Nd-YAG, 355 nm) laser; matrix – HCCA in ACN/aqueous TFA 0.1% (6:4, v/v) with the addition of N,N-dimethylaniline. MALDI MSI (pos): the 12 µm sections were obtained from the frozen bundle of Cuvierian tubules using cryostat microtome and placed onto an ITO-coated glass slide; Smartbeam (Nd-YAG, 355 nm) laser; matrix – HCCA in ACN/aqueous TFA 0.1% (6:4, v/v) with the addition of N,N-dimethylaniline; the spatial resolution was set to 60×60 mm²	PCA	[93]
H. atra, *H. leucospilota*, *Pearsonothuria graeffei*, *Actinopyga echinites*, *Bohadschia subrubra*	Profiling of triterpene glycosides in body walls and Cuvierian tubules of five tropical sea cucumber species	Animals were dissected into the body wall and Cuvierian tubules; organs were homogenized and extracted twice with 70% EtOH	Similar to [2]	LC-MS	RPLC: Symmetry C18 column (4,6×150 mm, 5 µm); gradient elution at 1 mL/min flow rate and 27 °C; A: H_2O; B: MeOH; run time: 15 min	-	[94]
					ESI-QTOF MS (pos): V_{cap} – 3.1 kV; cone voltage – 50 V; nebulization with N_2; desolvation temperature – 300 °C; source temperature – 120 °C		
				MALDI MS	MALDI-QTOF MS (pos): nitrogen laser (337 nm); matrix – HCCA in acetone		
H. forskali	Characterization of body wall triterpene glycosides, studying of	Relaxed animals were anesthetized in a 0.1% solution of 1-	Similar to [2]	MALDI MS	MALDI MSI: similar to [12] MALDI, ESI, and RPLC-ESI MS: similar to[11]	-	[95]

	their localization in the tissue, and investigation of the release of glycosides by the animal in its surroundings	phenoxy-2-propanol in seawater. Stressed individuals were mechanically disturbed by repetitive hitting. Cuvierian tubules from relaxed and stressed animals were collected and prepared for MALDI, ESI MS, and MALDI MSI. Extraction from the body wall and Cuvierian tubules was similarly to [11]					
H. scabra, *H. impatiens*, *H. fuscocinerea*	Characterization triterpene glycosides and comparison of body wall composition from three tropical holothurians species	Sea cucumbers were dissected and body walls from each species (5–7 samples) were homogenized and extracted twice with 70% EtOH	LLE: partitioned successively against *n*-hexane, CH_2Cl_2, $CHCl_3$, and *i*-BuOH; HPLC fractionation: C18 column (4.6 × 150 mm, 5 μm); gradient elution using 10% MeOH (A) and 100% MeOH (B)	LC-MS	nanoRPLC: C18 microfluidic chip with an enrichment column (40 nL) and analytical column (0.075×43 mm); gradient elution at 300 nL/min; A: 0.1% FA in 10% MeOH; B: 0.1% FA in MeOH; run time: 45 min<br>ESI-QTOF MS (pos)	-	[96]
				MALDI MS	MALDI-FTICR MS (pos&neg): neodymium-doped yttrium aluminum garnet laser (355 nm); matrix – 2,5-DHB in H_2O/ACN with the addition of NaCl		
H. scabra	Characterization triterpene glycosides in the body wall of *H. scabra* and comparison with glycoside composition of processed sea cucumbers	Three fresh animals were eviscerated and their body walls were homogenized in MeOH	Similar to [2]	MALDI MS	MALDI-QTOF MS (pos): nitrogen laser (337 nm); matrix – 2,5-DHB in H_2O/ACN with the addition of *N,N*-dimethylaniline	Non-parametric statistic Mann-Whitney test	[97]
H. sanctori	Characterization triterpene glycoside composition in the body wall and Cuvierian tubules of *H. sanctori*	Four fresh animals were dissected into the body wall and Cuvierian tubules and extracted with MeOH	Similar to [2]	MALDI MS	MALDI-QTOF MS (pos): nitrogen laser (337 nm); matrix – 2,5-DHB in H_2O/ACN with the addition of *N,N*-dimethylaniline	-	[98]
Eupentacta fraudatrix	Profiling, structural characterization, and studying of distribution	Seven fresh animals were chopped and subjected to the triple	SPE: BondElut C18, 100 mg/1 mL	LC-MS	RPLC: Zorbax Eclipse XDB-C18 column (1.0×150 mm, 3.5 μm); gradient elution at 0.1 mL/min flow rate and 40 °C; A: 0.1% FA in H_2O; B: 0.1% FA in MeOH; run time: 38	-	[99]

	of triterpene glycosides	extraction with EtOH; another seven animals were dissected into respiratory trees, body walls, gonad tubules, guts, and aquapharyngeal bulbs Separate organs were extracted with EtOH			min<br>ESI-QTOF MS (neg&pos): V_{cap} – ±4.0 kV; nebulization with N_2 at 0.8 bar; dry gas flow – 7 L/min at 200 °C		
H. scabra	Studying the chemical composition of triterpene glycosides released in surrounding seawater by *H. scabra*	*H. scabra* body wall were gently pinched to facilitate the release of compounds into a small volume (1 mL) of filtered seawater	SPE**:** Sep-Pak C18	LC-MS	RPLC: Poroshell 120 EC-C18 column (4.6×150 mm, 2.7 μm); gradient elution at 0.2 mL/min flow rate; A: 0.1% FA in H_2O; B: 0.1% FA in ACN; run time: 35 min<br>multimode source-QTOF MS	-	[100]
H. forskali	Investigation of composition and distribution of triterpene glycosides of *H. forskali*	Animals were dissected into body walls, gonads, and Cuvierian tubules and homogenized in MeOH	LLE: partitioned successively against *n*-hexane, CH_2Cl_2, $CHCl_3$ and *i*-BuOH	LC-MS	RPLC: Eclipse plus C18 column (4.6×100 mm, 3.5 μm); isocratic elution at 1 mL/min flow rate and 40 °C of 40% B; A: 0.1% FA in H_2O; B: ACN; run time: 15 min<br>ESI-QQQ MS (pos): V_{cap} – 3.1 kV; cone voltage – 40 V; source temperature – 100 °C; desolvation temperature – 300 °C nebulization with N_2 at 50 L/h for the gas cone and 500 L/h for the desolvation gas;<br>LC-IMS-QTOF-MS: V_{cap} – 2.5 kV; sampling cone – 40 V; source offset – 80 V; source temperature – 150 °C; desolvation temperature – 300 °C	-	[101]
				MALDI MS	MALDI-QTOF MS: Nd-YAG laser (355 nm); matrix – 2,5-DHB in H_2O/ACN with the addition of *N*,*N*-dimethylaniline		
H. leucospilota	Characterization of compositions and spatial distributions of triterpene glycosides in the body wall of *H. leucospilota*	Body wall extract: freeze-dried and grounded *H. leucospilota* body walls were extracted three times with 70% EtOH. Epidermis extract: pieces of the epidermal layer were separated from a	LLE: partitioned successively against CH_2Cl_2, EtOAc, and *n*-BuOH	MALDI MS	MALDI-TOF/TOF MS (pos): matrix – 2,5-DHB in 70% MeOH with 0.1% TFA;<br>MALDI-TOF/TOF MSI (pos)**:** carboxymethyl cellulose was used as an embedding medium of the body wall**;** the 20 μm sections were cut using cryostat microtome and placed onto an ITO-coated glass slide; matrix – 2,5-DHB in 0.1% TFA in MeOH/H_2O (7:3, *v:v*)	-	[102]

		thick dermis and extracted with H_2O or *n*-BuOH					
H. atra	Studying triterpene glycosides composition of *H. atra* by a combined approach including LC-MS/MS, Molecular Networking, pure compound isolation, and NMR spectroscopy	Fresh animals were chopped, freeze-dried, and extracted four times with EtOAc/MeOH (1:1, *v:v*)	LLE: partitioned successively against hexane, CH_2Cl_2, and butanol	LC-MS	RPLC: Kinetex PFP column (2.1×100 mm, 5 μm); gradient elution at 0.2 mL/min flow rate; A: 0.1% FA in H_2O; B: MeOH; run time: 33 min<br>ESI-QOrbitrap MS (pos&neg): spray voltage – 3 kV; capillary temperature – 280°C; sheath gas (N_2) flow rate – 40 unit; auxiliary gas (N_2) flow rate – 1.0 unit	Molecular Networking	[103]
Apostichopus japonicus	Characterization of the chemical composition of triterpene glycoside fraction from red and black types of *A. japonicus*	Sea cucumbers were dissected and body walls of different types of *A. japonicus* were extracted twice with 70% EtOH in a water bath at 90 °C	LLE: partitioned successively against hexane, $CHCl_3$, EtOAc, and *n*-BuOH	LC-MS	RPLC: Syncronis C18 column (2.1×100 mm, 1.7 μm); gradient elution at 0.2 mL/min flow rate and 35 °C; A: 0.1% FA in H_2O; B: ACN; run time: 40 min<br>ESI-QOrbitrap MS (neg): mass resolution – 70,000 FWHM; injection time – 200 ms	Student's *t*-test	[104]
H. polii, *H. leucospilota*, *H. atra*, *H. edulis*, *Bohadschia marmorata*, *Actinopyga mauritiana*	Metabolomic profiling and comparison of chemical compositions of extracts from the sea cucumbers inhabiting either Red or the Mediterranean Seashore	Sea cucumbers were dissected and body walls from each species (3 samples) were homogenized and extracted twice with 96% EtOH	LLE: extraction with a solvent combination of MTBE/MeOH/H_2O; the upper non-polar phase was re-suspended in *i*-Pro/ACN (3:7, *v:v*) and used for the analysis of the lipid content; the lower polar phase was re-suspended in MeOH and used for the analysis of the triterpene glycoside composition	LC-MS	RPLC polar fraction: ACQUITY HSS T3 column (2.1×100 mm, 1.7 μm); gradient elution at 0.4 mL/min flow rate; A: 0.1% FA in H_2O; B: 0.1% FA in CAN; run time: 17 min<br>RPLC non-polar fraction: Acquity BEH C8 column (2.1×100 mm, 1.7 μm); gradient elution at 0.4 mL/min flow rate; A: 0.1% Hac, 1% 1M NH_4Ac in H_2O; B: 0.1% Hac, 1% 1M NH_4Ac in ACN/iPro(7:3); run time: 17 min<br>ESI-Orbitrap MS (pos&neg)	PCA, heat maps and dendrogram representations	[105]
H. whitmaei, *H. hilla*, *H. atra*,	Profiling and characterization of the chemical composition of	Freeze-dried and grounded tissue samples were twice extracted	To analyze the content of crude extracts samples were dissolved in MeOH.	LC-MS	RPLC: Acquity BEH C18 column (2.1×50 mm, 1.7 μm); gradient elution at 0.6 mL/min flow rate and 40 °C; A: 0.1% FA in H_2O; B: 0.1% FA in ACN; run time: 28.5 min	Kruskal-Wallis test	[106]

H. edulis, *Bohadschia argus*, *B. vittiensis*, *Bohadschia sp.* *Actinopyga echinites*, *A. mauritiana*	anti-fouling extracts of sea cucumbers	with EtOAc/MeOH (1:1, *v:v*) followed by MeOH extraction	Sample Fractionation: LLE: partitioned successively against EtOAc/H_2O (1:1, *v:v*) and *n*-BuOH/H_2O (1:1, *v:v*); SPE**:** Supelclean LC-18, 60 mL/10 g; Semi-preparative HPLC: Pursuit XRs C18 column (10×250 mm, 5 µm)		ESI-QTOF MS (pos)		
H. scabra	Profiling and characterization of triterpene glycoside composition of the viscera of *H. scabra*	Dried viscera comprising the intestinal tube, the respiratory trees, and the gonads were ground and extracted with MeOH	LLE: partitioned successively against *n*-hexane, $CHCl_3$, CH_2Cl_2, and *i*-BuOH Fractionation by flash chromatography on a non-polar column (Büchi Cartouche FlashPure ID C18-WP Flash) followed by partitioning against isobutanol	LC-MS	RPLC: Acquity UPLC BEH C18 column (2.1×50 mm, 1.7 µm); gradient elution at 0.25 mL/min flow rate and 40 °C; A: 0.1% FA in H_2O; B: ACN; run time: 15 min	-	[107]
					ESI-QTOF-MS (pos): V_{cap} – 3.1 kV; sampling cone – 40 V; source offset – 80 V; source temperature – 120 °C; desolvation temperature – 300 °C		
				MALDI MS	MALDI-QTOF MS (pos): Nd-YAG laser (355 nm); matrix – 2,5-DHB in H_2O/ACN with the addition of *N*,*N*-dimethylaniline;		

Table S2. Instrumental and methodological details of MS-based applications for the analysis of starfish and sea cucumber lipids.

Species	Research focus	Extraction	Purification and desalting	Analytical approach	Separation and MS detection conditions	Statistical methods	Ref.
Acaudina molpadioides, Cucumaria frondosa, Apostichopus japonicus	Profiling and annotation of cerebrosides from three species of edible sea cucumbers	Dried sea cucumber body walls were homogenized and extracted four times with $CHCl_3$/MeOH (2:1, *v:v*).	LLE: extracted three times with EtOAc/*n*-BuOH (2:1, *v:v*); Column chromatography: silica gel column (solvent $CHCl_3$/MeOH/H_2O, 95:5:0 to 5:5:1, *v:v:v*) followed by separation on silica gel column with solvent $CHCl_3$/MeOH (25:1 to 10:1, *v:v*)	LC-MS	RPLC: TSK gel ODS-100Z column (2.0×50 mm, 3 μm); isocratic elution with acetonitrile/water (95/5, *v:v*) at 0.2 mL/min flow rate and 25 °C; run time: 30 min	-	[143]
					ESI-IT-TOF MS (pos): probe voltage - 4.50 kV; curved desolvation line and a block heater temperatures - 200 °C; nebulizing gas (N_2) flow rate - 1.5 L/min; drying gas (N_2) pressure - 0.1 MPa		
A. japonicus, Thelenota ananas, A. molpadioides, Bohadschia marmorata	Detection and structural characterization of cerebrosides from four species of sea cucumbers	Dried sea cucumber body walls were homogenized and extracted four times with $CHCl_3$/MeOH (2:1, *v:v*).	Dried extracts were saponified in 4 M KOH in methanol followed by LLE with $CHCl_3$/MeOH/H_2O; SPE: Supelclean ENVI-18, gradient elution using solvent $CHCl_3$/MeOH (100:0 to 0:100, *v:v*)	LC-MS	RPLC: Agilent Plus C18 column (2.1×100 mm, 3.5 μm); isocratic elution with 95 % MeOH with 5 mM ammonium acetate and 0.05 % HAc at 0.2 mL/min flow rate and 30 °C	-	[59]
					ESI-QTOF MS (pos): V_{cap} – 3.5 kV; nebulization with N_2 at 2.1 bar; dry gas flow - 5 L/min at 280 °C; sheath gas flow - 11 L/min at 280 °C; nozzle voltage - 500 V; fragmentor voltage - 135 V		
Pearsonothria graeffei	Investigation and identification of cerebroside molecular species from the *P. graeffei*	Dried sea cucumber body walls were homogenized and extracted five times with $CHCl_3$/MeOH (2:1, *v:v*).	Dried extracts were saponified in 4 M KOH in methanol followed by LLE $CHCl_3$/MeOH/H_2O SPE: silica gel column, gradient elution using solvent $CHCl_3$/MeOH	LC-MS	Similar to [28]	-	[144]
Cucumaria frondosa	Profiling, annotation, and investigation of the relationship between structure and activity of cerebrosides, ceramides, and sphingosines of sea cucumber *C.*	Cerebroside: dried sea cucumber body walls were homogenized and extracted by high-speed counter-current chromatography (HSCCC). Ceramide: sea cucumber body walls	Ceramides: LLE, three times with EtOAc/*n*-BuOH (2:1, *v:v*) followed by separation on silica gel column with solvent 95% MeOH	LC-MS (Cer.)	RPLC: Agilent Plus C18 column (2.1×100 mm, 3.5 μm); isocratic elution with 95 % MeOH with 5 mM ammonium acetate and 0.05 % HAc at 0.2 mL/min flow rate and 30 °C	ANOVA	[145]
					HESI-QOrbitrap MS (pos): ionization voltage - 3.5 kV; sheath gas - 30 arbitrary units; auxiliary gas - 10 arbitrary units; spray voltage - 3.5 kV; capillary temperature - 350 °C	-	

	frondosa	were homogenized and extracted three times with $CHCl_3$/MeOH/H_2O (4:4:1, *v:v:v*).		LC-MS (Sphing.)	RPLC: Agilent Plus C18 column (2.1×100 mm, 3.5 µm); isocratic elution with 98 % MeOH with 5 mM ammonium acetate and 0.05 % HAc at 0.2 mL/min flow rate and 30 °C<br>ESI-QQQ MS: nebulization at 40 psi; dry gas (N_2) at 350 °C; carrier gas flow - 8 L/min; fragmentor voltage - 200 V; collision energy – 30 eV		
Asterias amurensis	Profiling and characterization of cerebrosides from starfish *A. amurensis*	Whole bodies of starfish and organs, were extracted following the Bligh and Dyer method (extraction with $CHCl_3$/MeOH/H_2O)	Column chromatography: silica gel column eluted with $CHCl_3$/MeOH/H_2O (90:10:0.1, *v:v:v*)	LC-MS	RPLC: Inertsil ODS-3 column (4.6×250 mm); isocratic elution with 98 % MeOH at 0.9 mL/min flow rate<br>ESI-IT-TOF MS (pos): ion source gas (N_2) flow - 1.5 L/min; dry gas pressure - 155 kPa; interface voltage - 4.5 kV; curved dissolved line and heat block temperatures - 200 °C	-	[146]
				ESI MS	ESI-IT-MS (pos): ion source gas (N_2) pressure - 8.0 psi; dry gas flow - 5.0 L/min at 220 °C		
				MALDI MS	MALDI-TOF MS (pos): Nd-YAG (355 nm) laser; matrix: 2,5-DHB		
Parastichopus califormicus, C. frondosa, Isostichopus fuscus, Holothuria mexicana, H. polli, B. marmorata	Structural characterization and semiquantificatio n of the phospholipid profile of six sea cucumber species	Dried sea cucumbers were extracted following the modified Bligh and Dyer method (extraction with $CHCl_3$/MeOH/H_2O)	Extracts were redissolved in *n*-hexane/*i*-Pro (7:3, *v:v*)	LC-MS	NPLC: Nucleosil 100-5OH column (3.0×250 mm, 5 µm); gradient elution at 0.5 mL/min flow rate and 35 °C; A: 1% FA, 0.08% NH_4OH in *n*-hexane/*i*-Pro (7:3, *v:v*); B: 1% FA, 0.2% NH_4OH in *i*-Pro/H_2O (100:13, *v:v*); run time: 50 min<br>ESI-Triple-TOF MS (neg): capillary voltage of -4.5kV; nebulizing gas pressure - 50 psi; heating gas pressure - 50 psi; curtain gas pressure - 35 psi; heater temperature – 500 °C	Heatmap visualizatio n	[142]
B. marmorata, I. fuscus, H. polli, H. mexicana, C. frondose, P. califormicus	Structural characterization of the gangliosides of six sea cucumber species	Dried sea cucumber body walls were homogenized in H_2O followed by extraction with a solvent combination of $CHCl_3$/MeOH/H_2O	SPE: Supelclean ENVI-8 cartridge	LC-MS	HILIC LC: GOLD-amino column (2.1×100 mm, 1.7 µm); gradient elution at 0.35 mL/min flow rate and 40 °C; A: ACN; B: 5 mM ammonium acetate in H_2O; run time: 30 min<br>HESI-QOrbitrap MS (neg): spray voltage - 3.2 kV; capillary temperature – 250; aux gas heater temperature - 350°C	ANOVA	[147]

Table S3. Instrumental and methodological details in metabolomics studies of starfish and sea cucumbers

Species	Research focus	Analytical approach	Statistical methods used for treatment of analytical data	Ref.
P. pectinifera	Investigation the influence of different environmental factors such as water heating, oxygen deficiency, feeding, injury, and different water salinity on polar steroids of the starfish *P. pectinifera*	HPLC-QTOF MS	ANOVA, PCA, PLS-DA,	[87]
Apostichopus japonicus	Investigation the influence of different environmental factors such as heat, hypoxia, and combined stress on metabolome of the sea cucumber *A. japonicus*	UPLC-QTOF MS	Student's *t*-test and fold change analysis, PCA, PLS-DA, heatmap visialization	[171]
A. japonicus	Investigation the influence of acute hypoxia	UPLC-QTOF MS	PCA, PLS-DA, OPLS-DA	[172]
A. japonicus	Comparing the metabolic profiles of the thermotolerant strain and common *A. japonicus* under high temperature	GC-MS	ANOVA	[173]
A. japonicus	Metabolomic analysis of the coelomic fluids that were ejected by evisceration	UPLC-MS	fold-change analysis, *t*-test, PCA, OPLS-DA	[47]
A. japonicus	Investigation of alterations in *A. japonicus* metabolome during the aestivation stage	UPLC-QTOF MS	*t*-test	[174]
A. japonicus	Investigation of metabolic changes induced by skin ulceration syndrome	UPLC-QTOF MS	*t*-test, PCA, OPLS-DA	[175]
A. japonicus	Investigation of metabolomic differences between cage-cultured, pond-cultured, and bottom-sowing *A. japonicus*	UPLC-QTOF MS	*t*-test, OPLS-DA	[176]
A. japonicus	Investigation of *A. japonicus* coelomic fluids of different sexes and reproductive states	UPLC-QTOF MS	Student's *t*-test, and fold change analysis, PCA and PERMANOVA tests	[177]
A. japonicus	Investigation of metabolic differences in the muscle tissue of animals between the nonbreeding and growth stages	UPLC-QTOF MS	Student's *t*-test, ANOVA, OPLS-DA	[178]

A. japonicus	Comparing the metabolic profiles of four *A. japonicus* varieties (green, white, purple, and spiny)	UPLC-QTOF MS	*t*-test, PCA, OPLS-DA	[179]
A. japonicus	Investigation of metabolic changes in white, green, and purple *A. japonicus* body walls during the pigmentation process	UPLC-QTOF MS	*t*-test, PCA, PLS-DA, OPLS-DA	[180]
A. japonicus	Investigation of metabolic changes in *A. japonicus* of different geographical origins	UPLC-QTOF MS	*t*-test, PCA, OPLS-DA	[181]
A. japonicus	Investigation of melatonin-induced metabolomic changes in muscle tissues of sea cucumber	UPLC-QTOF MS	Student's *t*-test, and fold change analysis, PCA, PLS-DA,	[182]
A. japonicus	Investigation of metabolomic changes induced by pedal peptide-type neuropeptides	UPLC-QTOF MS	Student's *t*-test, and fold change analysis, PCA, PLS-DA,	[183]
